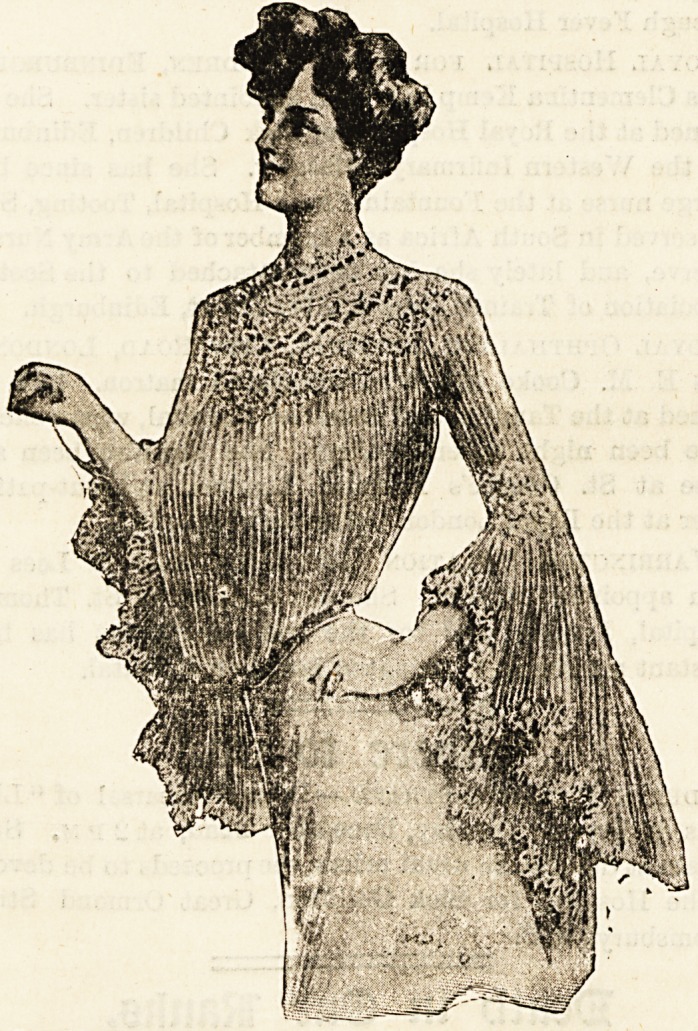# Nursing Section

**Published:** 1903-12-12

**Authors:** 


### The Hospital.

### Nursing Section.

Contributions for this Section of "THE HOSPITAL" should be addressed to the EDITOR, "THE HOSPITAL' NURSING SECTION, 28 & 29 Southampton Street, Strand, London, W.C

No 898.—VOL. XXXV. *SATURDAY, DECEMBER* 12 1903.

### Notes on News from the Nursing World.

### CHRISTMAS IN FOREIGN HOSPITALS.

THE special feature of the nursing section this week consists of the accounts that have been written for us, chiefly by nurses, of the manner in which Christmas is spent in some of the foreign hospitals. The description of the festivities at the Eppendorf Hospital, Hamburg, will be read with all the more interest because a rhyme recited by four little dwarfs was composed by two of the nursing staff. There, as in the Municipal Hospital at Berlin, a feature is made of the representation of the Nativity, and it will be observed that in Germany as in Norway Christmas trees are provided for both adults and children. Most of the arrangements in these hospitals appear, as in England, to be in the hands of the sisters. In the account of the Rigshospital one incident merits attention here. The shopkeepers of Christiana send toys, cakes, and chocolate from their respective establishments for the benefit of the children ; this example might be followed with advantage by some of the tradespeople in English towns. In Italy, of course, Christmas is but little observed, and though our contributor in Rome has sent illustrations of great interest, for which we are indebted to Dr. V. Gaudiani, it will be noticed that, while presents are given, the wards are not decorated and that there are no Christmas trees. It is gratifying to find that in our Government Hospital at Cyprus Turks co operate with Christians in decorating the wards for a festival in which, owing to the rules of their religion, they cannot participate.

### OUR CHRISTMAS DISTRIBUTION.

IT is possible that there may still be readers who have overlooked the latest date for sending in parcels for distribution at Christmas to the patients in hospitals and infirmaries. We therefore once more remind them that they must reach us at the latest on Monday next, in order that the whole of the articles received by us may be on view at the offices of THE HOSPITAL, 28 & 29 Southampton Street, on Tuesday afternoon, the 15th. Nurses who desire to see for themselves the garments which have been forwarded to us will be welcome any time between 3 and 5 P M. We have to acknowledge, with many thanks, a large parcel from Miss Evelyn M. Smith, 116 High Street, North Finchley, N.

### THE NURSES' CORONATION FUND.

A GENERAL meeting of the committee of the organisation called King Edward the Seventh's Coronation National Fund for Nurses in Ireland was held last week in Dublin It appears that Mr. Andrew Beattie presided, and that a certain number of the lady superintendents representing the nurses were also present. The meeting, it is stated, was called for the purpose of receiving the audited balance-sheet, for considering the provisional rules for the administration of the fund drafted by the special sub-committee and concurred in by the representatives of the nursing profession, and for the appointment of three trustees of the fund under the rules. But the figures of the balance-sheet are not mentioned in the newspaper report, nor are the rules set forth, though it is intimated that with some amendments they were adopted. A general meeting, however, is to be held on Monday afternoon next of subscribers and "of the nurses who shall have paid their annual subscription before the 12th instant," for the election of 15 members of the council under the rules of the society for the year 1904. We hope it is not true that nurses in Dublin are being pressed to join the Fund whether they wish to do so or not, and, as one of them puts it, "we dare not refuse."

### A COMPLIMENT TO IRISH ROMAN CATHOLIC NURSES.

THE Countess of Dudley last Friday was present at the annual meeting of the St. Lawrence Roman Catholic Home for Nurses, in Dublin, and took the opportunity of expressing the indebtedness of her committee for the establishment of district nurses in the poorest parts of Ireland, to the home, which, she said, had given great assistance in the carrying out of the undertaking. Her Excellency went on to observe that she could not speak too highly of the trained nurses whom the committee had engaged from the home. They had done their work in the most satisfactory manner, and they were exercising an educational and civilising influence among the people in the localities in which they were employed.

### THE ACLAND HOME AND DISTRICT NURSING.

WE understand that, owing to the increase of every branch of the work of the Acland Home at Oxford, it has been found desirable to divide the work of the district nurses from the Medical and Surgical Home. The few simple rules which were made in 1879, when the institution was established, are now, of course, entirely unsuitable for the conduct of the greatly increased sphere of work, and a special committee has therefore been appointed in order to draw up fresh rules and a new constitution. It is expected that the report of this committee will be issued to the subscribers about February.

### THE LADY SUPERINTENDENT OF PRINCE ALFRED HOSPITAL, SYDNEY.

MISS S. B. MCGAHEY, who, in consequence of ill-health, has just resigned the post of lady superintendent of Prince Alfred Hospital, Sydney, which she has occupied for 12 years with great success, mentions in some notes on the progress of that institution that a preliminary training school, where suitable candidates will be received and trained for a certain number of months, is likely to be started at an early date. Miss McGahey has also resigned the honorary secretaryship of the Australasian Trained Nurses' Association, which she has held for more than four years. The present membership of the association is 621 general and 147 midwifery nurses, and Miss McGahey's official services have been extremely valuable. At the annual meeting she was presented, on behalf of the members, with an association badge in gold, suitably inscribed, and a spray bouquet, as a token of their appreciation of her work.

### THE SUSPENSION OF A NIGHT NURSE AT TRIM.

A remarkable inquiry has just been concluded in the board room of the workhouse at Trim by Dr. Joseph Smyth, an inspector of the Irish Local Government Board, concerning the suspension of Miss Margaret J. Sheridan, night nurse in the union infirmary. Pending the issue of Dr. Smyth's report it would not, of course, be fair to comment on the evidence. But the issue turns upon whether Miss Sheridan was, or was not, justified in refusing to take charge of the consumptive patients at night. The nurse, who was trained at the Mater Misericordiæ Hospital in Dublin, admits that she declined to undertake that particular duty, for which she was not engaged, so long as the Guardians would not put up a covered passage from the infirmary to the consumption wards which have been recently erected. She contends that she was entitled to adopt this attitude as a means of compelling the board to effect a necessary and inexpensive improvement.

### THE REFUSAL OF A CORRECT CERTIFICATE.

Apparently the authorities of the Guest Hospital at Dudley do not intend to send Nurse Gallagher a correct certificate. We are satisfied that, whatever may be the cause of this extraordinary attitude, their refusal cannot be justified There is only a choice of two courses in such cases One is to decline to grant a certificate at all, and the other is to give it in accordance with the custom of the hospital. To give a certificate which is only half correct reflects discredit on the givers.

### THE RESULT OF A RASH EXPERIMENT.

It is a matter for regret that an organisation founded by the late Bishop Lightfoot, with the best possible intention, for the most praiseworthy object, should be in low water. But the fate which threatens the Bishop Auckland Nursing Association can hardly be said to be unexpected. A year ago it was converted into a provident society, and, as a local paper tersely puts it, "the new scheme does not seem to have worked well." Another, and perhaps more potent reason, is that the Bishop Auckland and District Workmen's Association, which secured the services of Miss Wood when she left the original organisation, is increasing in popularity. We did not anticipate that the Bishop Auckland people would be able to maintain the association, and we think that the discontinuance of one will probably mean enhanced support for the other.

### DISTRICT NURSING IN CHELTENHAM.

At the annual meeting of the Cheltenham District Nursing Association the report and accounts were adopted. The former showed that the number of visits by the general nurses had increased to the extent of more than 2,500, and those by maternity nurses upwards of 1,000. The speakers at the meeting vied with each other in their praise of the nurses, but the financial state of affairs is not healthy, the income being £59 less than the expenditure. An interesting feature was an address by Mrs. Percy Boulnois, who, as a stranger, specially congratulated the association upon providing maternity nurses. Canon Roxby, rector of Cheltenham, said that he could not conceive of any agency in town or country more useful to the poor; and after this and other testimony to the value of the organisation, it may be hoped that next year the financial position will be as satisfactory as the work accomplished.

### MALE NURSES AND THE ROYAL ARMY MEDICAL CORPS.

The assertion of "A Trained Male Nurse" a fortnight ago that the training under the auspices of the Royal Army Medical Corps is better than that of the National Hospital for the Paralysed, has naturally provoked protests from those whose experience has been gained at the institution in Queen's Square. We think that a correspondent this week makes a very good point. He asks why, if the training of the Royal Army Medical Corps orderlies is a complete one, should they go to the National Hospital for a training? As four probationers are at the Hospital at the present moment who have had military experience, one having been upwards of six years in the Royal Army Medical Corps, the value of its training for men who desire to be proficient nurses is attested by the persons who are most able to appreciate it.

### ACTION FOR LIBEL BY A HANDSWORTH NURSE.

At Birmingham County Court on Friday last an action was tried in which the plaintiff, a nurse, sued Mrs. Anderson Thompson, of Amberley Ridge, Stroud, to recover damages, £300, for libel. It was stated that the plaintiff had been nursing Mrs. Thompson but was recalled by the matron of the Handsworth Nursing Home, by whom she had been sent. Application was made to the defendant for two guineas due to plaintiff, and Mrs. Thompson thereupon wrote to the matron of the home declining to pay, and saying that the plaintiff had disgraced herself by her conduct. This was the libel complained of, and the defence was that the letter was privileged and that it was not of the libellous character alleged. The hearing of the case occupied two days, and the jury being then unable to agree, were discharged.

### THE BETTER WAY.

Last week we mentioned the purchase of a piano by the Christchurch Guardians for the nurses of the Union Infirmary. At Lanchester the superintendent nurse made an application for a similar gift. Instead of the matter being discussed by the Board, however, Mr. Logan, one of the members, generously offered to make the Infirmary a present of an instrument; and the offer was, of course, gratefully accepted.

### The Nursing Outlook.

" From magnanimity, all fear above;
From nobler recompense, above applause,
Which owes to man's short outlook all its charm.'

### THE POOR POOR-LAW NURSE.

For many weeks our columns have harboured letters pointing out the injustice of not recognising infirmary training at its full value; the injustice is not only to the nurse but to the patient, and the whole subject of the position of the Poor-law nurse is in such a parlous state, that we wish to call the special attention of our readers to it. Of course the proposal of the Local Government Board to introduce a sort of semi-nurse of one year's training was the first attack; then came the decision of the Midwives Board that the midwives under the Poor-law and in infirmaries were exempt from all the rules of Section E—that is to say, all the rules with regard to disinfection, case-taking, care of infants' eyes, and so on. This second blow has not received the consideration it deserves, for it shows how the feeling is growing that second-rate things are good enough for infirmary nurses, and that it is no use to try and keep up the status of the Poor-law nurse, or to secure the best nursing for the pauper patient. Then came the series of appointments of hospital-trained nurses as heads of infirmaries, though the applicants included many women thoroughly trained in Poor-law work.

Evidently we must not let our sword sleep in our hand at present or we shall have all the good work done by Miss Twining and others lost, and our workhouse patients being cared for by imbeciles and all the horrors of the old days back with us once more. It is strange since we have such training schools as Birmingham and Crumpsall and Marylebone, and such excellent matrons as heads of infirmaries, and such much better buildings and appliances than in the old days, that we should have this swing-back in workhouse nursing. There is really no better training to be secured anywhere than in some of our English infirmaries. The probationer has to sign for three years; she has her lectures and classes and examinations; she is paid a good salary, and in many ways her life compares favourably with that of a hospital nurse. Certainly the training is better and the comfort is greater in a workhouse infirmary than in most of the small hospitals that ask a premium from their probationers. If there are not quite enough applicants for vacant posts this is solely due to the foolishness which refuses to recognise the infirmary as the best possible training school for those who mean to remain in Poor-law work. The majority of the Guardians have done their best for the comfort of the nurses and for the training of the probationers: witness the charming home just opened at St. George's-in-the-East; witness the tennis and croquet lawns at Kingston. There ought to be no brighter and no more efficient branch of the profession than the Poor-law service; it is work that calls out all a woman's sympathy for the poorest and weakest, and it is a field with great opportunities in the future if only the present evil time can be tided over.

And happily this is a question in which we can all stand shoulder to shoulder. There has been no dissentient voice in the nursing world to the principle that the Poor-law nurse should be in training and status on a footing with all other nurses. For it has been felt everywhere that if once the "qualified" nurse of one year's training were recognised, the result would be the degradation of all those who hold the title of nurse, and the whole profession would suffer. And we can all do something in the fight; we can all help to form public opinion; we can all declare in season and out, that we believe firmly that the sick pauper needs as good nursing as the sick prince, and that the work of nursing the one is as onerous and as honourable as that of nursing the other. Of course, the guardians and the infirmary superintendents, and the leaders of the nursing world generally can do most, and they must not let the question be forgotten: they must go on fighting. And they must watch well, for jeopardy seems to be on every side; the very least slight to an infirmary training, the smallest attempt to overlook the claims of the Poor-law nurse, must be a point of battle just now.

While our enemies are at the work of destruction it would also be worth while for us to do some work of construction. An effort to remodel those infirmaries which are behind the times, the drawing up of a scheme for linking small infirmaries to large for purposes of training, an effort to secure good probationers and to demand their very best in return for thorough training and good living conditions—all this would be useful. Just now in the rush of Christmas work it is easy to forget the professional side of nursing, but the present danger is too serious to be safely put aside, and we hope to hear of further meetings and resolutions against the lowering of the status and training of the Poor-law nurse before Mr. Walter Long finally decides which of the recommendations of the Departmental Committee he proposes to adopt.

### Christmas in Foreign Hospitals.

### The Eppendorf Hospital, Hamburg.

The Eppendorf Hospital, situated just outside Hamburg, is the largest in Germany, and is considered a model one. The foundation-stone was laid in 1885 and the opening took place in 1889. In 1901 it was enlarged. It is built on the pavilion system and consists of 95 separate buildings, 66 of which are pavilions for the patients. It is like a small town in itself with its Strassen (streets), and Marktplatz (market-place) in the centre. The site is a splendid one and commands a magnificent view over the city of Hamburg and the surrounding country. The grounds are beautifully laid out and afford pleasant walks for the inmates. The south-west front faces a charming little park belonging to the hospital. It is much frequented on Wednesdays and Sundays (visiting days), by the visitors, for whose accommodation special trams run from Hamburg. The hospital is capable of containing about 2,000 patients, and, taken all together, doctors, nurses, servants, workmen, etc., there are 760 persons employed in it. Electric light is laid on everywhere. The hospital is entirely under the control of the director, Herr Professor Lenhartz, who is widely known and respected as a great authority in the medical profession.

The Eppendorf Hospital, Hamburg.

After this little introduction I will proceed to describe the festivities at Christmas. These generally take place about December 22nd, and begin at 6 P.M. The most interesting are in Pavilion 6, which is devoted to the children. It is a very pretty sight. Everything is done to make it look as festive and as like Christmas as possible. From the ceiling hang graceful festoons, rows of lighted candles are placed in the windows, and best of all, at any rate in the eyes of the children, are the Christmas trees, four in number, which stand down the middle of the room, making a perfect blaze of light with their innumerable candles. In the spaces between the trees are tables covered with all sorts of wonderful toys and presents of books and clothing, etc, each marked with the name of the lucky little owner. Mounting guard just inside the door is a venerable old snowman, armed with a most formidable-looking stick, whose awe-inspiring appearance, I may add, is greatly enhanced by a top hat! Just behind him is a Punch and Judy show, Punch and Judy looking saucily out at everyone and appearing quite ready to take their share in the fun. It is a great pleasure when there are numerous empty little beds to be seen, showing that many little patients are well on their way to recovery.

There are the children, a joyous group in the middle of the room, arrayed in different costumes, some as angels, some as dwarfs, and others in snowy-looking garments representing snowflakes. At 6 o'clock the visitors begin to crowd in, among them the worthy Herr Pastor in his black gown and Elizabethan-looking ruff. The said black gown one year sadly compromised the dignity of our friend the snowman by brushing his top hat from his head, greatly to the delight of Punch and Judy, who had been peeping round the corner, trying to poke fun at him.

When everything is ready, the following specimen pro-

### CHRISTMAS IN THE EPPENDORF HOSPITAL, HAMBURG—*Continued.*

gramme is gone through:—A carol, by the nurses; a recitation, by one of the little angels; a carol, by the children. Then comes an address by the Pastor, followed by a recitation by the Snowflakes. After which two small persons advance and sing "Süsser die Glocken nie Klingen." One takes treble and the other alto, and it really is beautiful. As they sing we can easily imagine we are listening to the voices of angels come down from Heaven to teach us the wonderful Story of the Nativity. When it is finished, four little dwarfs appear and recite an amusing rhyme, written by two of the nurses. It is so funny that I have translated it and tried to put it again into rhyme, as follows:—

First Dwarf.

Good evening, ladies and gentlemen all,
The happy Christmas feast did us call
From our forest home so far, far away.
The path was quite easy—that we must say;
For Pavilion Six is so very well known,
In Fairyland e'en, as all must own.
Have you heard of the beautiful Forest Queen,
Whose throne is the prettiest e'er was seen?
Of silver and branches of fir 'tis made,
And when the roses are blooming, she said
She'd invite you all to her beautiful bower,
And charm you all with her magic power.
And a special kind greeting I had to bring
To Uncle Professor,* of doctors the King
If there's aught that we want, we've only to ask—
To procure it for us he makes it his task.
From morning till night for many a year
He's cared for sick children, to whom he's so dear.
And to please them at Christmas he always brings
These toys and the other wonderful things.
From our hearts do we thank him everyone
At this happy time for what he has done.
(*Then turning to Herrn Professor Lenhartz*)
We wish you all happiness, joy and peace
And showers of blessings that never may cease.
We dwarfs in the forest will most careful be
To keep you and yours from all danger free.
For everyone honours you, both great and small,
Dear Uncle Professor. And now we will call
For a right hearty cheer from everyone here
Who to join us has come to our feast this year.
(*All together*) Three cheers for Uncle Professor—Hip, hip, hip, hurrah!

Second Dwarf (*bustling up as if he had not a moment to spare*)

I'm in a great hurry and can't stay to rest
So much Christmas shopping is really no jest—
So much to do—it makes me quite dizzy,
I'm like Dr. Kissling, who's always so busy.
For e'en in the forest we've heard of his fame,
And how you all love but the sound of his name.
For to the sick children he's always so kind—
His equal, I'm sure, is not easy to find,
And although his hours of leisure were rare,
For us children he'd often have time to spare.
We love him so much, how much we can't say.
And when he left us, it was a sad day.

Third Dwarf.

Uncle Dr. Reye, the excellent man,
Laughs with us and cheers us as much as he can,
"Children, how lucky you are, you don't know,
To be cared for and tended and treated so."
So says he, and then oft must leave us, you see,
For Uncle Professor's right hand is he.
As doubtless you know, Uncle Dr. Preyss
Was also once here He was so nice.
We shall never forget him, you may be sure,
He gave us oft chocolate. That was the cure
We liked best of all. He'd also a knack
(Out of sheer love to us) of giving a smack!
That didn't matter. Good order must be!
And the chocolate tasted fine, you see!

Fourth Dwarf.

But we must not forget Dr. Renz to mention,
Who pays to us always so much attention
And makes for us children so very much fun.
"Father Christmas" he's called by everyone.
And last but not least Dr. Wagner is here,
Who's just been appointed to us—this last year.
We love him right well and will do what we can
To please him, for he's a most hardworking man.

First Dwarf.

And now as we've farther to go this night,
To make our adieux we think it but right.
But ere we depart, our duty is clear
To give hearty thanks to our Christmas guests here.

All Four Dwarfs.

Farewell! Farewell! We must away,
And hope to meet you a year to-day.

The Dwarfs' recitation is followed by two carols. While these were being sung the sliding doors at one end of the pavilion, leading out into a sort of verandah, are slowly pushed back and reveal a living picture of the Holy Child in the stable at the inn in Bethlehem. In the background are two figures representing Joseph and Mary, the latter bending over the manger in which lies a real baby. Outside stand two angels. The whole is illuminated by a subdued red light. It is very impressive, and there is a hush over the whole room. The doors are drawn together again, and when they re-open disclose the same scene, but this time three figures representing the Wise Men from the East bow before the manger in an attitude of adoration. It is all beautifully and reverently done, and one cannot help feeling how forcibly the truth of the Nativity must be brought home to the children by having it represented to them in such a real way. It would, I think, be impossible for anyone who had seen it to forget it

The programme being now ended, the children are able to look at and admire their presents. It is a pretty sight to see them. One wee mite attired in pale blue gauze is toddling along wheeling a doll's perambulator almost as big as herself, with the utmost gravity and importance. Another poor little thing, too ill to get up, is sitting in bed with her toys spread out before her. As I passed her by I said, "Well, little one, are you pleased with all your nice things?" She said nothing but looked up with a smile of unutterable bliss. And what need had I of further answer?

The festivities in the other pavilions are very similar, that is to say, the decorations are much the same, there are Christmas trees in all and presents for the patients, but of course there is no programme gone through. As there are about 1,700 patients in the hospital at Christmas time, the preparations for such a celebration are no light undertaking.

* Nearly all German children say "Onkel Professor," "Onkel Doktor," and so on.

### The Rigshospital, Christiania.

The porter of the Rigshospital opens the great double gates and sledge after sledge drives in through the lofty portals. A strong odour of moss and resin, in fact the smell of the great forest itself, is accompanied by some fifty fresh pine trees, pervading the atmosphere generally only visited by poor human creatures with whom life has dealt hardly and who often bear the impress of death.

The sledges stop outside each of the great white brick buildings of the hospital and the trees are carried up the wide staircases into the wards of the different departments, the large trees going to the large wards and the small ones to the more modest rooms. None of the sick people will be without them, all must have the greeting from outside—from the country and the forest—and all must see children, and had sent money to the sisters, and now the lads were to have a new jacket and the little girls new pink petticoats and pretty pink silk ribbons in their hair. But how would it all come to pass if they had to lie in their beds for ever? Here was the second great surprise. They were to get up to dinner, all who could possibly move. Three of them were generally able to walk about where they liked, but on that evening 20 beds were to be vacant. Only five children in that ward had to remain in bed on the Christmas Eve, but even they were not so sorry about it for they were so ill and so tired that they could not even think of getting up. There was no doubt about it all, for the sisters had said so, the doctors had said so, and the professor himself had proclaimed it in a loud voice so that all had heard it at the same time.

Exterior of the Rigshospital, Christiania.

the lights glimmer between the green branches like the stars that twinkled on the first Christmas night. But the tallest and most beautiful trees are carried into the children's great ward, for those trees are intended for the little ones.

The children lie in their cots and then the trees are brought in. Those who can, sit up in their beds and gazed at them; some cry for pure joy, and others again remain quite still and only gaze and gaze. It is all so real to them!

For a long time before the children had chattered about Christmas amongst themselves, and the sisters had had to tell them again and again how beautiful it was all going to be. It began with the splendid news that they were going to have new clothes, every one of them. People out in the town and all over the country had thought of the sick little

The busiest of all was the sister, who had to take charge of the presents. The professor, the doctors, the students, all sent money, and a collection was also made at the office. The Botanical Garden sent flowers, and shops all round the town sent toys, the confectioners contributing cakes and wonderful figures in chocolate and marzipan. The whole town wanted to have a share in the Christmas joy of the sick children.

The other departments of the hospital also had their plentiful share of presents which were to be distributed amongst the sick, to those who needed them most, and who knows this better than the sisters? The entire staff has to work harder now that it is Christmas, but even they are to have cakes, apples and sweets in plenty. All shall feel that it is Christmas and nobody shall feel forgotten or abandoned in the hospital at that happy time.

### CHRISTMAS IN THE RIGSHOSPITAL, CHRISTIANIA—*Continued.*

And Christmas Eve is coming. The Christmas trees are green no longer, almost hidden as they are by a glitter of gold and all the colours of the rainbow. Between the branches there shines golden fruit and twinkling stars, and the further the gaze of the children travels, the more wonderful are the things it meets. But far away in a large heavy basket there lies something towards which they cast eager glances. They cannot help it, for it is such a wonderful basket. It is quite covered by a thin transparent coloured cloth, a cloth so light that it looks like rosy, green and blue mist, and under this mist they can distinguish a multitude of white packages, both large and small, and many of mysterious shape. The sisters are there, the doctors are there, and even the professor. Then all the lights are lit, the chaplain reads out the gospel of Christmas and utters a prayer whilst the sisters take the youngest children in their ring. Yes, if we look closer we find it is a wonderful children's party we have joined!

The climax of the feast comes when the great basket is brought forward. The professor says in a loud voice that he wants two children to come forward; one of the biggest boys and the strongest girls. But of course none like to step out of their own accord, for who would like to proclaim himself as being big and strong? But the sisters pick them out and then they come and stand in front of the professor. It is truly necessary that they should be big and strong, for there is a heap for them to do. The light-coloured gauze is drawn to one side, and the professor takes out each parcel and calls out the inscription. "Little Oscar." A little girl immediately runs with the parcel to Little Oscar, who must answer "Yes" in a loud voice, for that is what the professor said they were to do.

Children's Ward, Rigshospital, Christiania.

laps so as to keep them quiet a little while longer. The children are so anxious to be good, so anxious to hear what is said, and to join in the prayer—but how can they, when all the lights are shining so brightly on the trees? But still they can sing when the time comes to join hands and to walk 'round the trees. It hardly goes as quickly as at an ordinary children's party, for even though the eyes shine brightly from sheer pleasure and the cheeks glow from the motion, there are many who find it difficult to move freely. One has still got an open sore in his back, another is tired, oh so tired, from many months spent in bed, and others again suffer from St. Vitus's dance. One of them has some trouble with his head, it insists on nodding and nodding in all directions whilst the face makes the most weird grimaces. Another little one has it in the legs. They will not follow reason but go their own wild way, so that it is difficult to join in the "Thorwold of E!" Now it is a boy's turn, and he has to run off. "Sister Nogot!" "Bergliot!" "Little Johanna!" "Dr. Frolich!" There are even parcels for the professor, and when he has to call his own name the children are delighted!

There is a life, a movement, a running to and fro, and a showing of toys and sugar figures, shouting and joyous laughter. But away in a quiet room there may be seen two parents bending over a little cot. They dare not leave their child for a moment, for it only has a few minutes to enjoy their presence before going far away where "there is no sorrow, no sickness, and no death."

Outside all the church bells are in harmonious cadence ringing in deep and solemn tones. They call to each other and answer each other as if the words ring through the air :—

"Glory to God in the highest'

### Berlin Municipal Hospital.

" Silent night—Holiest night,
All asleep—lonely night,
Where adoring watched the pair,
Watch as in His Father's care,
Sleeps the infant Christ."

Thus had they already sung some time before Christmas in the different pavilions of the large municipal hospital at Friedrichshain, in Berlin. This hospital is built upon a large piece of ground with handsome garden-parterres, and consists of 15 pavilions, with 950 beds for the sick, and various administrative and other buildings. The municipality provides the money for the Christmas festivities, part of which comes from kindly benefactors. In every pavilion there is a head sister, with whom are associated three or four sisters, male and female nurses as may be required, and one house servant. Bustle and activity begin to rule the day from the moment when the head sister becomes possessed of the means with which to make the Christmas purchases for outside matters—such as fetching provisions, keeping the corridors and stairs clean, filling the ice-press, etc.—becomes at these times an extremely important personage; indeed, he is almost exalted into being a minister of the interior, for not only does he select the fir trees, but he also supplies the larger ones with candles, which he lights at the time of the festival. Then he stays beside them, as long as they are burning, in order to nip in the bud all possible danger. With the smell of the fir trees there is soon mingled a sweet odour of gingerbread; nuts and apples also will be forthcoming in abundance, all sent by the managers of the hospital.

On Christmas Eve the sisters are busy in their own pavilions until far into the night, decorating the long tables and jointly apportioning the gifts for every single patient. Besides these, provided with a liberal hand, are many coloured plates of sweets, and everyone receives useful and enjoyable presents.

Exterior of the Municipal Hospital, Friedrichshain, Berlin.

her patients at her own discretion. In the children's pavilion, above all, the utmost joyful excitement is to be found. For weeks before the festive season the industrious sisters instead of, as at other times, going early to bed wearied by their labours, all sit down together for hours at a time. Their clever fingers, which by day bind up so many wounds and cool so many fevered brows, are now actively engaged in dressing dolls, and renovating all possible little articles for fitting up shops and such like. They also arrange little verses and peformances which the children are to recite at the festival. Some days before Christmas the indoor servants procure the gigantic fir trees. In each of the large wards containing 30 to 40 beds, stands a stately tree, reaching from floor to ceiling. For each smaller room and extra room small trees are prepared, for there is not one poor patient in the Friedrichshain who on that blessed night does not gaze with delight upon a Christmas tree. How joyfully all the patients look up when the green tree is brought into the ward! How spicily it smells and diverts the mind from its own sorrows to think upon the day which brought salvation into the world, the Feast of Love. The indoor servant, who at other times is only entrusted with

And at last Christmas Day has come. The whole morning intense excitement and activity bear sway; all tasks are performed extra well and extra quickly. The patients are given clean bed-linen and clothing, so that they may be quite smart for the festival and the following holidays. As darkness comes on the well-rehearsed song is practised once again, the patients who are allowed to be up group themselves in a semi-circle around the tree. From that time solemn expectant silence reigns. Without a word the tables of gifts are carried in, the candles on the tree are lighted, and on the entrance of the honoured guests the song is struck up. The guests consist of the councillors and managers of the hospital, the pastor, and their families. After the song the sisters give out the presents. The astonishment and delight in the faces of the recipients is a real joy to behold. One who came there, ragged and shirtless, now sees before him a fine woollen shirt, warm stockings, and a brush and comb, so that he may practise the cleanliness he has learnt in hospital. An old grandmother gazes with much emotion at the beautiful hood and thick shoes, how warm they will keep her ears and feet! Shortly before the festival began, a poor girl, suffering

CHRISTMAS IN A BERLIN MUNICIPAL HOSPITAL—*Continued.*

from rheumatism in the joints, was brought into the room and put to bed amid cries of pain. And now, although she is a stranger in this gathering, she sees by the light of the Christmas candles a sister coming smilingly towards her, and actually an expensive woollen coat and warm gloves are given to her! Already she anticipates how they will protect her in the future from a similar severe chill. She is wonderfully cared for within the walls of this house.

The guests now pass from one pavilion into another. The children have the greatest festivities; scarcely is the door of their pavilion opened before we hear the young voices singing:—

"Holiest night, on angels' wings
Dost thou softly approach the world."

After the song the pastor addresses the gathering. Suddenly, from beneath the brilliantly-lighted tree the figure of a child-angel steps forward, and, as the spirit of the Christmas tree, speaks a few words and then the ever-sweet children's song breaks forth:—

"Oh, fir-tree, Oh, fir-tree, how green are thy leaves."

But we are as curious as the children and hardly have leisure to listen to the various pretty little performances that follow; our eyes are irresistibly drawn from all the splendour before us to where, half under the little tree, is the manger with Joseph and Mary and the Holy Child in all pious simplicity. In the background stands a stately doll's house, in which even grown-up's can look round and by stooping may enter. It is furnished with every comfort and the doll-folk who are sitting at table are really to be envied. A charming carriage stands before the hall door drawn by a pair of goats nearly life-size; in the carriage sit two most elegant baby dolls. There stands a mighty castle that is stormed from above by the enemy, and here we really see our dear old fairy tales personified before our eyes. There is Little Red Riding Hood in the wood, looking for flowers; near her is the wolf, and there we see little Snow White behind the mountain, the seven dwarfs taking care of her. They look quite captivating—the little brown gnomes with their long beards and the fair-haired Snow White. It is quite easy to enter into the Prince's love for her.

As yet untouched, the shops are ranged in fresh glory. Pots, cushions, and sugar-loaves, all are well supplied. "Alas! how soon doth form and beauty vanish away!" we quote forebodingly.

"Uncle Doctor, please, please," cry the little ones joyfully; and Uncle Doctor, who is so dreaded when dressing wounds and so beloved in playtime, is violently dragged off to the doll's house; there he must give a performance, and he knows his duty far too well to omit a representation of Punch.

Woman's Ward, Municipal Hospital, Friedrichshain, Berlin.

How many a poor child comes here, as they do in thousands, and in one night is altogether transformed into a new life by the sight of the wonderful things, and when he goes out again later on, and returns—as alas! so many do—to dirt and uncleanliness, yet his stay in the hospital ever remains with him as a bright memory. There reigned peace, joy, order, and beauty.

"Oh! thou joyful and blessed Christmastide that bringeth mercies!" So say we all out of full hearts, when we see joy beaming in so many eyes and so many trembling hands outstretched in gratitude.

### Christmas and the Roman Hospitals.

To the Eternal City must be accorded the distinction of possessing probably the most ancient hospital in the world. Long before the mighty Dome of St. Peter's rose in majestic grandeur, towering above every other building in Rome, the Hospital of San Spirito carried on its benevolent work under the shadow of the Castle of St. Angelo.

Historical records state that it was built as a Hospice for the Saxons by their King Ina in the year 717 A.D., and called "Scola Saxonini."

Thus even in those warlike times, some provision was made for the sick and infirm, rough and inefficient as it must necessarily have been.

who took the direction of its affairs for a considerable period, but finally it has received the name of San Giovanni from its vicinity to the Basilica of St. John Lateran, the original metropolitan church of Rome.

Built upon one of the seven hills of Rome—Monte Celio—the hospital is healthily situated, and is within a short distance of the Great Coliseum. It was enlarged to its present size by Pope Alexander VII., and then assumed its modern aspect, but traces of the original building continue to exist in its structure. There is provision for 500 beds for women, besides a certain number for children over 7 years of age. According to recent regulations no infectious cases

Students at the Operation Table, Hospital of San Giovanni, Rome. Professor Mazzoni Performing the Operation.

Hardly less important and noteworthy is the Hospital of San Giovanni, the largest hospital for women in Rome, which was founded in the year 1216 by Cardinal Giovanni Colonna, a member of the highest Roman nobility. It is interesting to note that the head of the family of Colonna has the privilege of standing at the right hand of the Pope during a Papal Mass, and it was the present Prince Colonna who, in his capacity as Syndic of Rome, received our own King Edward when he recently visited the Eternal City. The Hospital of San Giovanni was erected near the ancient church of St. Andrew which still exists, and was at first named after that saint; in later days it was known as the Hospital of San Salvatore, from a confraternity of that name are admitted. About twenty years ago, under the influence of the prevailing theories of modern hygiene, the hospital was completely renovated and so thoroughly transformed as to make it quite up-to-date and worthy of its renown as one of the most thoroughly equipped hospitals of modern Italy.

The wards are large, lofty, and imposing, with tiled floors, and walls and bedsteads all painted white. The beds are covered with white quilts, and as the patients all wear white garments a general air of purity and cleanliness prevails without the touch of colour one usually sees in the wards of an English hospital. Ventilation is well regulated, but as the windows are often placed near the ceiling, the patients are not distracted by seeing what goes on in the outside

CHRISTMAS AND THE ROMAN HOSPITALS—*Continued.*

world. Twice a week visitors are admitted, and then the great wards are filled by a multitude of dark-complexioned, picturesque Italians, who come to visit their friends and relations. It is not unusual to see three, or even four, rows

Small Ward, Hospital of San Giovanni, Rome.

of beds in the largest wards, as no patients are turned away if it is possible to find room for them; but as ample cubic space is provided and there is plenty of fresh air, no feeling of closeness is perceptible in the atmosphere.

At Christmas time, special treats are provided for the patients by different members of the committee, usually in

Long Ward, Hospital of San Giovanni, Rome.

the form of useful gifts for the adults and toys for the children; but beyond fresh flowers for the altar, and an extra supply of candles, no decoration of the ward takes place. As is usually the case in Italian hospitals, the menial work of the wards is done by women of the servant class, who wear a white overall but no head-covering. The responsibility of carrying out medical instructions rests with a sisterhood, founded in 1821 by the Princess Teresa Doria Panfili, for the express purpose of undertaking the nursing of the hospital. These religious sisters wear black dresses, flowing white aprons, and a very quaint black net cap made upon a wire frame, resembling in shape the early Victorian bonnet. The sisters, who number more than eighty, administer medicines, take temperatures, make bandages, have the care of the instruments, distribute the food, supervise the clothing and bed-linen, and overlook the patients generally.

Four large wards are devoted to medical diseases, and one very long ward to surgical cases especially connected with the diseases peculiar to women. Every department is under the care of from three to five physicians or surgeons, who wear long white coats all the time they are on duty in the hospital. The operation rooms have stone floors, white-washed walls, and glass ambulance tables. The operation tables, one need hardly say, are constructed according to the latest hygienic principles, and the floors are thoroughly cleansed and disinfected with bounteous supplies of carbolic between each operation. A special operation room is set apart for laparotomy, which room is very frequently white-washed. Aseptic surgery is very completely carried out in the hospital of San Giovanni.

The surgical department is under the direction of Professor Mazzoni, one of the most celebrated surgeons of

A Foundling at the Hospital of San Giovanni, Rome.

Rome and indeed of all Italy. He became well known some years ago by an entirely successful operation which he performed on the late Pope; but he is best known by his brilliant statistics of operative surgery, a work which is a source of great pride to all Italian surgeons. Professor Mazzoni is greatly loved and admired by medical students who come from all parts of Italy to witness his operations.

The writer enjoyed the distinction of being personally conducted through the wards of the hospital by the eminent Professor himself, and of witnessing a very serious operation of exceptional interest to an English nurse.

Apparently the religious sisters are never present in the operation room, perhaps the rules of their Order do not allow it.

With such grand opportunities for training probationers, one cannot help wishing that the time may not be far distant when the hospital of San Giovanni will have its own training-school for nurses, who will themselves become capable heads of wards in the future. The difficulties in the way of realising this desirable change can, however, hardly be imagined by those unacquainted with Italian ideas of etiquette and propriety. However greatly an Italian lady may desire to study the art of nursing, she will very rarely find it possible to enter a public hospital where alone such knowledge is to be acquired.

The photographs accompanying this article were obtained through the kind courtesy of Dr. Vincenzo Gaudiani, who also supplied valuable information respecting the hospital, of which he is a member of the staff.

### Christmas at a Government Hospital in Cyprus.

How amazed the old crusaders, who landed here centuries ago under Richard the Lion-heart on their way to Palestine to fight to the death against the Mahommedans, would have been could they have looked forward and seen us as we appear at Christmas time! Christians and Turks all happily helping to decorate wards and corridors together in preparation for the Christmas-tree. Our head dispenser, a strict young Turk, is our great stand-by on these occasions, for his height gives him a great advantage when branches and palms are required to be fixed in rather inaccessible places, or candles on the topmost branches of a tree nearly reaching the ward ceiling.

We are busy decorating for two days before the tree is to be shown so as to get it done, for we nurses want Christmas Day free. Downstairs are piles of palm-branches, eight or 10 feet in height, pepper-tree branches, with their long trailing leaves, oranges with their own stems and leaves, large oval Jaffas, and sweet, tiny mandarins from the Abbot of Kikko's garden, lanterns of all kinds and colours. Upstairs, in our sitting-room, are dozens of coloured and muslin bags, being filled with sweets and biscuits, parcels being tied up with ribbon and labelled, toys chosen for each child, and, to show it is a Government hospital, red-tape, after bothering us metaphorically all the year, is being made useful for once in its life by helping to tie everything at all heavy on to the tree.

The tree itself when finished looks lovely—a beautiful straight pine, reminding us of our happy summer-house on Troödos amongst the bracken and pines where now on the hills we can see the gleam of Christmas snow. It is so bright and sparkling with its pretty ornaments and candles, Father Christmas with his long white beard, and a lovely supply of presents for everybody. It is planted in a large tub draped with red and pepper-tree branches. The ward jalousies are twined with lemons and oranges, and lanterns hanging over each bed give quite sufficient light to the room.

Christmas day is our day. We go in the morning to the service in our little English church, very pretty with palms and roses and great white datura-bells on the font. A full church and a bright service; all the dear, well-known hymns; then the Christmas greetings in the sunny little churchyard. After a quiet afternoon with just an eye on things in general, and the decorations still in progress in entrance-hall and corridor we go out to dine with friends. It is a dark evening and we have an orderly with a large lantern in front, though we have only a hundred yards or so to go, for there have been several people attacked and robbed lately, and our head native nurse cannot bear the thought of our venturing out alone at night, though really the people would never attack anyone English; it is only their own countrymen whom they dare to molest. Well, we have a real Christmas evening—a blazing pudding, with all sorts of treasures, ring, thimble, button, etc., hidden in it. Of course we drink to "Absent Friends," and end up the evening playing games with our friends, children, and music; getting home after all quite safely.

Next day the hospital children can hardly keep their excitement within bounds all the morning. They have a tea, of course; cakes, Turkish delight, nuts and oranges; with the exception of two little convalescing typhoids whose goodies have to be saved up for them. Then when 5 o'clock really strikes and they hear the Government House carriage drive up, and see all the little English children with mothers and nurses arriving, they realise that the happy moment has really come. The Cypriot children are quiet little things, and do not give vent to such shouts of delight as the little English boys and girls; but oh! how bright their faces are, and how they hug their dollies and their Noah's arks, or blow sly notes on their trumpets and look half afraid of the delightful noise they make! One little boy, who had his hand so crushed by investigating a steam roller too closely that the arm had to be amputated below the elbow, is cheerfully pulling crackers with the other hand. He is a little rascal, and his accident has not in the least cured him of his mischievous tricks. Our ovariotomy case is able to come in from her special ward, and is delighted with her warm shawl from the tree. The doctor is surprised by a basket wonderfully made out of cardboard and gelatine paper by one of the native nurses, containing new laid eggs from her own white hen. In fact everybody seems to have exactly what he or she wanted, and the children are so excited and pleased that not one wants to go to sleep. One tiny stone-boy gives his treasures to nurse and begs her to take care of them in her own room; he is so afraid they might disappear before morning.

At last it is all over and Bairam has begun. There will be throwing the jereed in the moat this afternoon. All the Zaphteihs riding madly up and down throwing the long, slender jereeds at each other. Men, women and children all dressed in the gayest and brightest of garments crowding the ramparts above to look on at the show. A pink and yellow brocade jacket, white loose trousers, red and blue stockings of a most wonderful pattern, sash of all colours, and a red fez, being nothing out of the common. Next week the Greeks will have their turn and start their festival by going to church at 3 A.M. Our poor nurses and orderlies cannot share any of our good things at Christmas (patients of course are exempt from fasting), but we have saved a turkey for them, and after the New Year dance at Government House some of the pretty things left over are generally sent to the Hospital in time for their feast, after which we settle down to everyday life again.

### "The Hospital" Convalescent Fund.

The Hon. Secretary begs to acknowledge with thanks a contribution of 5s. from Nurse M. Barnet Groom for the above Fund.

### The Evolution of the Modern Nurse.

BY AN OCCASIONAL CORRESPONDENT.

In order to trace the connection of woman with the art of healing, one must go back to a very early period indeed, for both in ancient Egyptian and Grecian mythology, according to tradition, female deities occupied important relations to the science of medicine. To Isis, the ancient Egyptian goddess of medicine, who was the wife of Osiris, the Egyptians attributed peculiar medical skill, for she is said to have restored her child Horus to life. She is also the reputed discoverer of several remedies, which even as late as the time of Galen bore her name, and the herb vervain credited down to the Middle Ages with magical properties, was known as the "tears of Isis." Hygeia, who was said to be the daughter of Asklepios, the Grecian god of medicine, was worshipped in the temple of Argos as the goddess of health, both physical and mental. The Greeks also ascribed medical skill to Juno, who, under the name of Lucina, was held to preside over the birth of children, and was probably the first presiding deity of gynæcology. Ocyroe, the daughter of the centaur Cheiron, was also said to possess the power of healing. The story of the birth of Moses illustrates the important part played by women in gynæcology at that early period. There is a Grecian bas-relief that depicts a woman binding up the wounded limb of a soldier, which evidences the fact that her gentle touch was early recognised in administering to the sick. Homer also makes allusion to the woman's part in nursing the warriors who were wounded in battle. Dunglison states that the wives of the Druids, who were called "Abravnes," exercised the calling of sorceresses, and tended to wounded soldiers.

### The First Female Practitioner.

The first female practitioner of medicine who received a medical education and training appears to be Agnodice, a young Athenian lady who lived about 300 B.C. To satisfy her desire for knowledge, she donned male attire, and braving the fatal results of detection, attended the schools of medicine which were rigidly restricted to men. On completing her studies she determined to keep to her disguise, and commenced to practise medicine in the Grecian capital, her efforts speedily meeting with success. She made herself a specialist in the diseases of women. The physicians of Athens soon became jealous of

A Nurse of the Fourteenth Century Giving Food to a Patient.

her reputation and rapidly increasing practice, and summoned her before the Areopagus and accused her of abusing her trusts in dealing with female patients. To establish and prove her innocence she then disclosed her sex, on which her persecutors accused her of violating the law prohibiting women and slaves from studying medicine, but the wives of the most influential Athenians, much to their credit, rose in her defence, and eventually obtained a revocation of the law.

### A Predecessor of Florence Nightingale.

In a Roman lady, named Fabiola, we find an early predecessor of Florence Nightingale, for she is held in grateful remembrance as the founder of hospitals in Italy, and is said to have personally nursed the sick at Ostia. She was of the illustrious house of Fabius, which was celebrated in the fourth century for piety and charity. The establishment of hospitals is commonly attributed to the Emperor Julian about the year 362 A.D., with whom Fabiola was contemporary. Celsus, who wrote in the time of Augustus, mentions large hospitals where patients were treated with specific medicines. The nursing of the sick as an actual calling, however, arose with Christianity, for it was about that period, between 400 and 500 A.D., that special institutions were founded for the care of the sick, and with them came the demand for a special class of persons who were expected to search out and convey to these institutions those who required medical treatment. Besides these ordinary professional nurses, male and female, there were also the pious people who voluntarily devoted themselves to the care of the sick.

### Special Orders of Nurses.

After the Crusades special orders of nurses developed mainly under the influence of the great religious houses. Such were the Brothers of St. Anthony, the Alexians, the Beguins and Beghards, the Black Sisters, the Lollhards, Cellites, Lazarists, and Hospitallers; Sisters of St. Elizabeth

A Nurse of the Fifteenth Century Feeding a Patient of St. Catherine, of Christian Love, of our Blessed Lady Sisters and Brothers of Charity, and the Ladies of St. John Some of these orders have survived many centuries and are in existence to-day. The statutes of the Hospital of St. John of Jerusalem, which is probably the most ancient, took their origin in the year 1181. Many of these early institutions for aid to the sick were in private dwellings, similar to the private hospitals of to-day. In process of time, however, there arose separate houses, usually connected with the churches and monasteries, where the sick were received and tended. Leper hospitals existed in every European country, for the need of isolation for sufferers from that terrible disease was recognised even in those days. Besides such institutions, there also existed at a very early period, special hospitals, whose sole object was the reception and treatment of the sick. From the eighth to the twelfth century, under the influence of Mahomedan rule, women were placed in excessive isolation, and it is not surprising to find under these circumstances, that certain women became skilled in attending to the medical requirements of their own sex. Albucasis of Cordova, one of the most skilful surgeons of the twelfth century, employed the services of properly instructed women for assistance in operations on females. Avicenna, a great physician of the Middle Ages, writing of remedies for diseases of the eyes, mentions a lotion compounded by a woman well versed in the medical art.

### Nursing by Nuns.

In Christian countries the monks and nuns nursed and tended the sick as an act of charity and piety. In the fifteenth century nurses were also common in private houses. Abelard permitted the practice of surgery to those of the convent of the Paraclete, over which Héloïse presided. Some of the abbots and abbesses attained a considerable proficiency in medicine and botany, and their names have come down to us in connection with works they wrote on these subjects. Hildegarde, the abbess of the Convent of Rupertsberg, near Bingen on the Rhine, in 1098 A D. wrote a book dealing with herbs and simples, called the "Book of Health," which had great popularity for centuries. Among others who devoted thenselves to the care and nursing of the sick were Radegoude, of France, the founder of a convent at Poictiers; the pious Elizabeth, of Hungary, who died in 1231; and Hedwigia, wife of Henry the bearded. In the famous school of Salernum, which was founded by the Benedictine monks in the eleventh century, women played an important part, and many were engaged in the preparation of drugs and cosmetics and in the practice of medicine. Such were Abella, who was the author of two medical poems; Costunza Calenda, the beautiful daughter

A Nurse of the Sixteenth Century.

of a skilful physician, who graduated in medicine; also Mercuriade, who wrote several medical treatises; Marguerite of Naples, Rebecca Guarna, Adelmota Maltraversa, and Irotula of Ruggiero, wife of a physician, who practised medicine and had a world-wide reputation. In the armies, however, the soldiers often perished for lack of attention, and the only nursing they got was from the baggage women—a disreputable class who followed every army into the field.

### The Order of Midwives.

In the fifteenth century midwives received instruction in their calling from the priests and monks, who in some places even superintended their work. There were five midwives in the city of Würzburg in the year 1450, and it appears from the city archives that they were compelled to attend the poor and rich by day or night. If they wished to go into the country they were obliged to give notice to the Burgo-

Midwifery Nurses at a Confinement in the Seventeenth Century.

master. In case one alone at any time could not terminate a labour, she was allowed by the law to call in others, on which occasion, however, they were not allowed "to scold, stop or swear at each other." The fees at that time, which were also regulated by law, did not err on the side of liberality, for the nurse was recompensed with 4 schillings, which is about sixpence for each case. It is satisfactory to know, however, that they were allowed to take more if it was offered. In the sixteenth century both male and female nurses were to be found in almost every city, and many of these, as the natural result of the Reformation, were now lay persons. The hospitals, however, still remained in the hands of the religious orders.

### Anna Morandi.

In concluding this brief sketch of the evolution of nursing, a subject which is worthy of further attention, mention must be made of that remarkable woman Anna Morandi, who attained a distinction that has never since been equalled by any member of her sex in the study of anatomy. She was born in Bologna in the year 1716, and married one Giovanni Manzolini, a maker of anatomical models in wax. She assisted him in his work and under his instruction gained an intimate knowledge of anatomy. Encouraged by Galli, a famous surgeon and professor of gynæcology at Bologna, Anna began to lecture on anatomy to private classes. Her skill in dissection requiring great delicacy of touch was phenomenal, and so clearly was she able to demonstrate the structure of the body that she soon acquired a universal reputation and her lectures became frequented by students from all parts of Europe. Shortly after the death of her husband in 1755 she was appointed to the chair of Anatomy of her native city, a distinction which had never previously been conferred on any woman. She was the first to reproduce in wax the capillary vessels, and her collection of models is still preserved in the city in which she laboured and spent her life. She died in 1774.

### Association for Promoting the Training and Supply of Midwives.

On Wednesday last week, a conference was held at 2 Cromwell Houses, by kind permission of Mrs. Samuel Bruce, to consider methods and means for promoting the training and supply of midwives. The chair was taken by Lord Monkswell, and among those present were Viscountess Knutsford, Lady Mary Glyn, Sir Michael Foster, M.P., Mr. Skewes-Cox, M.P., and Dr. Downes of the Local Government Board.

The Chairman said that he came there to learn, aud would like to know whether the Association held a strong opinion as to the Midwives Act being administered by a central authority, or whether they thought it better for it to be under the jurisdiction of the borough councils. The General Purposes Committee had received petitions from 17 borough councils in favour of the administration of the Act being in their hands, and were so much impressed by this concurrence of opinion that they passed a resolution stating that, subject to certain conditions and restrictions, the Act should be administered by the borough councils. Latterly, however, the General Purposes Committee, showed signs of a desire to rescind this resolution.

### Statement by Mrs. Bruce.

Mrs. Wallace Bruce, on behalf of the executive committee of the Association, then made a statement showing the work done by it towards securing the passing of the Midwives Act. Now that they had succeeded in achieving this object they found that their work had only begun, for there was no indication in the Act showing how or where the training of those who were to take the places of the superseded midwives should be done. Their first duty then was to organise a supply of trained midwives. A large army of midwives were now practising, but few of them had sufficient means to obtain the necessary training. The requirements of the Board as to training were high, and this increased the necesssity for assistance towards this training. The livelihood to be gained by midwives was so small that it was impossible to expect that women could afford to spend twenty guineas upon their training. The need would have to be met by voluntary effort. The Association hoped to act as a national general council for the training of midwives, and aimed at co-operation with all the associations at present at work in the counties. They had already a bureau established whence information could be obtained, and the next thing was to appeal for funds. They must all realise that a very large sum would have to be forthcoming from the whole country. Grants or loans would have to be made towards the training of suitable women. Those who had a free training would be bound to serve under the Association for a certain number of years. They hoped that they would receive aid from local associations and also from the Technical Education Boards. They were very anxious the training should only take place in good hospitals or well-known institutions. They had already opened a small training home at East Ham.

### The Importance of Supervision.

Miss Amy Hughes, Superintendent of County Associations, affiliated to the Queen Victoria Jubilee Institute for Nurses, then opened the discussion. After briefly describing the work done by the County Associations in rural districts, she went on to say that when the lines upon which the Institute was worked were first laid down the midwifery question had not come into so much prominence, but the Institute soon felt that in the country districts they must endeavour to prevent all work of that nature falling into the hands of those whose only knowledge was what had been handed down to them from their mothers and grandmothers; county associations were therefore started in connection with the Institute to meet this need. All their nurses were not fully trained unless it was necessary, but all were midwives. It was found in rural districts that the birth-rate was not high enough for the nurses to be kept solely as midwives, so that they also obtained training in elementary home nursing. The great point she wished to emphasise was that they were not allowed to work without strict supervision. That must also be the keynote of this Association. It was incorrectly imagined that proper training for midwifery could only be had in the London lying-in hospitals, but there were excellent means of training in the country towns. Her two main points were the necessity of funds for training the right kind of women and the vital need there was that they should have expert supervision. It would be many years before the reproach that English midwives were the most ignorant in Europe could be removed.

Miss Broadwood, of the Affiliated Benefit Nursing Association, was of opinion that the Association was aiming a little too high, and should be content to begin on a small scale. The cities should be the training grounds for midwives, especially those of the working classes who could go into the country and eke out their living in various ways.

Mrs. Rickman, of the Rural Midwives Association, was strongly of opinion that mothers ought to be allowed a choice of midwives, and she also deprecated the combination of district nurse and midwife.

### The Medical Standpoint.

Mr. Shirley Murphy, Medical Officer of Health under the London County Council, said that when he first looked at the Midwives Act he came to the conclusion that it might be entitled an Act for the Prevention of Midwifery, because if the rules were enforced a very small proportion of the existing midwives would be able to comply with them. Unless an organised effort was made for the supply of midwives numerous cases would occur when the poor women would be unable to obtain midwives. The Association should do nothing to pauperise the women, and should define the circumstances under which aid might be given to the training of midwives. He thought that, in view of the fact that this Act regulates the practice of midwifery and creates a dearth in the supply of midwives, the County Councils should be asked to contribute towards their education.

### Midwifery and Nursing.

Sir Michael Foster, M.P., said that the great object of this Association was to replace as speedily and as completely as possible the present midwives. Although they might set up a very high standard for the midwives of the future to reach at present this must not be too exacting. He wished the regulations were less stringent. What they had to provide more especially was a supply of midwives for county districts, so that a poor woman would not have to go out of her own village for a midwife. The midwives in villages could not earn more than a very limited income, and therefore they ought not to require an equipment out of proportion to their future gains. He considered it most undesirable to mix up nursing with midwifery, but saw no objection to the midwife having lodgers or a shop. The training of midwives should not be confined to any great centre, but adequate training should be provided in every large town.

### Offer of Scholarships.

Miss Wilson, President of the Midwives Institute, member of the Central Midwives' Board, said that the Association aimed at doing a splendid piece of work. In 1905 there would be an enormous want of midwives. How was it to be met? Abroad this system had been at work for 20 to 30 years. What they must do was to choose women who would be best fitted for the work, who would be able to form a sound judgment as to when to send for a doctor, etc. For the encouragement of the Association she informed them that the Institute had received an offer from an anonymous donor of a number of scholarships on condition that the training was absolutely thorough.

The following resolution was then proposed by Miss Clifford, Poor Law Guardian, Bristol:—

That this meeting is warmly in favour of the work and aims of the Association for Promoting the Training and Supply of Midwives, and will do what is possible to further the object of organising the training of midwives on a sound basis.

The resolution was seconded by Lady Mary Glyn, and was passed unanimously.

Lord Monkswell then repeated his question as to the authority by which the Act was to be administered, and Mrs. Wallace Bruce replied that the Association was warmly in favour of the administration being under a central authority, and was against its being delegated to the borough councils. A resolution to this effect was put to the meeting and—with one dissentient—carried.

### Nurses' Missionary Union.

An interesting meeting was held at the Church Home of All Souls', Langham Place, last week, in connection with the Nurses' Missionary Union, which has recently been formed to interest members of the nursing profession in medical missions in foreign lands, to help them to take their share in this work, and to serve as a bond of union between those nurses who desire to become missionaries or are in any way interested in this movement. About 140 nurses, representing a large number of hospitals and infirmaries, besides many who are engaged in district and private nursing, responded to the invitation. The upstairs room of the Church Home was prettily arranged, and here each nurse was warmly welcomed by the Rev. F. S. and Mrs. Webster. Dr. G. W. Guiness then gave an excellent address on the great need of medical missionary work in China, and Miss K. Miller, Secretary of the Nurses' Missionary Union, a short account of its nature and aims and also of its progress during the last few months. She mentioned the pressing need for nurses in mission hospitals abroad, and showed how the Union seeks to be the link to bring into touch with the missionary societies those nurses who have the desire to devote their lives to this work. She pointed out that one of the chief aims of the Union was to help nurses during their hospital course to begin to fit themselves for the work by encouraging and helping them to form little missionary associations among themselves in each hospital for united bible and missionary study. The proceedings concluded with a stirring address from the Rev. F. S. Webster.

### Presentations.

Royal Hospital for Sick Children, Edinburgh.—Miss Chafram, the assistant matron of the Royal Hospital for Sick Children, Edinburgh, left on November 30th to take up her duties as matron of the Children's Hospital, Birkenhead. She was presented with a silver afternoon tea service, on an oak tray, by the matron and nursing staff; and with a set of silver teaspoons and a pair of sugar-tongs by the domestic staff.

### To Help the Nurses to Help Themselves.

**Royal National Pension Fund for Nurses.**—The past year has been in many respects the most noteworthy in the Fund's history, for it has witnessed the issue of the third valuation report, which is even better than its predecessors. Not only policy-holders but all interested in nurses will be gratified to learn that the profit earned in the course of the five years ended December 31st, 1902, was two and a-half times greater than during the previous quinquennium, and amounted to over £27,900, of which £26,000 has been distributed amongst members for the purpose of increasing their annuities, irrespective of bonuses from other sources. Over 900 policies were issued, a number considerably in excess of any previous year, not excepting 1902, which has hitherto been considered the "record" year. The sick pay distributed amounted to £1,730, while at the last quarter of the year pensions and bonuses were being paid at the rate of over £10,000 per annum. The income was £120,000, and the invested funds exceeded £800,000—these are impressive figures. Offices: 28 Finsbury Pavement, London, E.C.

**The Junius S. Morgan Benevolent Fund.**—This is an auxiliary to the Royal National Pension Fund for Nurses. It was founded by nurses as a memorial to the late Junius S. Morgan, and raised to handsome proportions by the Morgan family and many other friends to nurses. An influential committee (many members of which are hospital matrons) supervises the investigation of claims, and devotes much care and attention to the relief of necessitous members of the Pension Fund. Secretary, Mrs. Bretland Farmer.

**"The Hospital" Convalescent Fund.**—The object of this fund is to provide rest for weary and needy workers during convalescence after illness amidst suitable surroundings, without any anxiety about ways and means. Since its establishment many tired and delicate nurses have enjoyed a much-needed change of air such as they could not possibly have secured for themselves without help. Experience has proved that it is better to let the nurses have a choice of locality rather than to send them to one settled place, and nurses are accordingly sent to all parts of the country. Contributions which would increase the field of usefulness are invited by the Hon. Secretary, care of the Editor of The Hospital.

**Queen Victoria's Jubilee Institute for Nurses.**—The Institute trains nurses in district nursing, and supplies nurses to affiliated associations for the sick poor in their own homes. Applications for information should be addressed to Miss Peter, the General Superintendent. The offices are at 120 Victoria Street, S.W.

**East London Nursing Society.**—The object of this society is to nurse the sick poor in East London in their own homes by means of trained resident nurses, each nurse living in the parish in which she works. In 1902 the staff of 29 nurses attended to 4,103 persons, to whom 103,809 visits were made. Annual subscriptions and donations to the general fund are asked for. Secretary, Mr. Arthur W. Lacey, 43 Rutland Street, New Road, Commercial Road East, E.

**The Colonial Nursing Association.**—This valuable association was founded seven years ago to supply trained nurses to the Crown Colonies and small British communities in foreign countries. Since the foundation 192 nurses have been despatched to various parts of the world, grants in aid being made where it is clearly shown to be impossible for the residents unassisted to bear entire cost of passage monies, salaries, and maintenance. The Hon. Secretary, Mrs. Debenham, Imperial Institute, S.W., will be glad to receive contributions, especially as an effort is being made just now to extend its benefits to the poorer colonies.

### Christmas Books.

FIRST NOTICE.

We have received a number of Christmas books for notice, and our readers will find them arranged below in a manner which we hope will aid them in their choice. The name of each book, its author, publisher, and price are given, so that at a glance can be seen all that it is necessary to know before ordering. Children's books, as usual, appear in larger number than others. The following, all marvels of cheapness, with excellent illustrations, binding, and letterpress, are from Messrs. Nelson and Sons.

### BOOKS FOR CHILDREN.

"Our Dogs" (1s.) is a charming picture-book containing coloured sketches of dogs at home and dogs abroad which will delight the little ones. "The Book of Horses" (1s ) is equally good, and the coloured illustrations are delightful. "The A.B.C. (of Games and Toys)" (1s.) is a pretty novelty in alphabets, and "The Doll's House" (6d.) will charm little girls with its quaint pictures. Pretty books can also be had for the modest sum of threepence. "Crackers" (3d.) and "Stories for Sunday" (3d.) are both good specimens of illustrated picture-books at this price. "Boris the Bear-Hunter," by Fredk. Wishaw, illustrated (1s. 6d.), gives thrilling descriptions of the perils and escapes which Boris encountered when bear hunting in Russia. "The Gorilla Hunters" (1s.) is a reprint of an old favourite, by R. M. Ballantyne, illustrated. "The House on the Moor," by Harold Averay (1s.), is a story of school boys and school life, illustrated. "The Round Tower," by F. M. Scott and Alma Hodge (1s. 6d.), is an Irish story of the rebellion of '98, illustrated. "Daddy's Lad" (1s. 6d.) is a pretty story of a charming little lass, with a picture of herself as frontispiece, by E. L. Haverfield; coloured illustrations. "Isabel's Secret" (2s.), a girls' story, suitable for Sunday reading, by the author of "The Story of a Happy Little Girl." "A Fair Jacobite," by H. M. Poynter (2s. 6d.), is a tale of the exiled Stuarts. The scenes are laid chiefly at St. Germain, and the book is one which will please girls who like historical stories; coloured frontispiece. "For King or Empress" (3s. 6d.), by Chas. W. Whistler, is also a historical romance. It deals with the state of England at the period following the siege of Oxford by King Stephen, when the power of Empress Matilda was on the wane. It is written in an interesting and graphic manner. "The Castle of the White Flag" (5s. 6d.), by E. Everett Green, illustrated. This tale of the Franco-Prussian War is brightly written, and will interest young readers of either sex. The story is told by various members of the Seymour family who found themselves unexpectedly in the midst of the great war of '70. They were detained in France, in consequence, in a château for a year. The illustrations give a graphic idea of the striking scenes and incidents in which they took part.

### MACMILLAN AND CO.

"Three Rascals," by Raymond Jacberus (4s. 6d.), illustrated. This volume relates the escapades, capitally told, of two small boys and their sister when they go to stay with an old Scotch housekeeper, of the friends they made, of the adventures they met with, and the scrapes they got into and out of. It is just the book for small boys and girls of nine or ten. "The Children Who Ran Away," by Evelyn Sharp (4s. 6d.), is a charming child's book. Prue and her brother Ricky, finding the monotony of nursing life at the top of a London house quite unbearable, decide to run away "into the real country where you can see every bit of the country without the houses getting into the way." How they carried out their attempt and its results is prettily and naturally told.

### MR. ANDREW MELROSE.

From Mr. Melrose we have received some well-got-up books. Lovers of cats may be interested in the story of "Pussy Meow," the autobiography of a cat, by S. Louise Patterson, illustrated (2s. 6d.). It is designed to teach children kindness to animals, "particularly to a much maligned and misunderstood fellow-creature, and to secure for her the consideration which humanity owes to the dumb."

"Girls Together" is a charming story for older girls, by Louise Mack (Mrs. T. P. Creed) (3s. 6d.), illustrated. It tells of the home life, friendship, and ambitions of two girl students. "Irene," by the same author, is for younger readers (3s. 6d.), illustrated. It is a story of Australian school life, which girls on this side will find very interesting.

### WARD LOCK AND CO., LIMITED.

Among the books received from Ward, Lock and Co. is "Betty and Co.," by Ethel Turner (3s. 6d.), illustrated. It contains some good short stories, reprinted from various magazines, which would be very good for reading aloud to the young ones.

The following books are new editions of old friends:—

"Tom Brown's School-days" (1s.). "East Lynne" (1s. 6d.), by Mrs. Henry Wood, illustrated. "The Head of the Family" (1s. 6d.), by Mrs. Craik, illustrated. "John Halifax" (1s. 6d.), by Mrs. Craik, illustrated.

### CASSELL AND CO., LIMITED.

A very useful little book which is sure to have a ready sale is, "How to Get up a Children's Play," by Maggie Brown (6d. net). The directions are so clearly given throughout that everyone should have a copy of the little book. It will be invaluable to persons getting up children's plays.

In the same series, "Little Folks' Plays," are "Rumpelstiltzpin and Dummling" (6d.), and "Cinderella" (6d ), adapted for small children's performances. Each play has coloured illustrations of the characters in costume.

### WELLS GARDNER, DARTON AND CO.

"Lollipop Town" (1s.), simple stories in large type and short words. This is a wonderful shilling's worth of pictures, poems, and short stories for the wee ones.

"Bert's Holiday." By Janie Brockman (1s ). Illustrated. A story of how Bert spent his holidays in the country with his cousins instead of at school, and of the fun and frolic he had there with them.

"Ragamuffin Tom." By J. E. Partridge (1s ). Illustrated. At the end of the book we read that Tom's end was a satisfactory one. "Ragamuffin Tom still lives; we shall not say where. He is an honoured citizen now, but he has never forgotten his early life, nor ceased to be thankful to the friends who gave him his first start."

"Constable Stories." By Flora Schmalz (1s.). Illustrated. It will amuse boys, and has a realistic picture of a policeman intercepting a robber as frontispiece.

"Black Polyanthus and Widow Maclean." By Jean Ingelow (1s.). An illustrated reprint of two excellent stories.

(*To be continued.*)

### Novelties for Nurses.

BY OUR SHOPPING CORRESPONDENT.

### KING EDWARD'S CHOCOLATES.

THIS is the name given to a delicious selection of Cadbury's chocolates, which are encased in most artistic boxes to form suitable Christmas gifts. To my mind no firm excels Messrs. Cadbury in the good taste displayed in the designs for their boxes. Added to this, that their chocolates and sweetmeats are of the very highest excellence, their commodities should be much sought at this time of the year especially. There is no excuse for buying foreign chocolate, considering that Cadbury's chocolate is no more expensive, and is of the highest quality and absolute purity. It can be obtained from every reliable vendor of sweetmeats.

### CHARMING BLOUSES AT PENBERTHY'S.

MR. PENBERTHY has recently extended his business at 388, 390 and 392 Oxford Street, and now not only gloves, fans, and other accessories of dress can be bought there, but a large selection of blouses, for both day and evening wear, is on view in the show-rooms. With these I was particularly pleased, and I should like to call attention to the one illustrated in the accompanying block. It is an evening blouse, suitable for "at homes" or theatres, made of accordion-pleated chiffon, in a variety of pretty shades—black, blue, pink, green, mauve—and having fancy lace and velvet yoke. It falls into particularly graceful folds, and, considering the amount of work in it, the price (25s. 9d.) is not an exorbitant one. These blouses are very useful for the nurse who discards uniform when dining with her friends. The popular golf blouse or jersey, hand-knitted by Scotch peasants, in blue and other colours, has been improved in shape, and can be worn either single or double-breasted. Pocket-handkerchiefs, always acceptable as Christmas presents, are in great variety, from dainty hem-stitched lawn, embroidered with a corner initial and spray, to the elaborate Maltese or Luxembourg lace, enclosing a minute square of silk. Three and elevenpence will buy a real Maltese lace handkerchief, while the initialled hem-stitched ones referred to just now are from 6s. 6d. a dozen. Mr. Penberthy's gloves are well known, but I should like to specially mention a new glove, imitation doe-skin, which wears admirably. It is spliced at the thumb in the same way as its counterpart in doe-skin, and is a first-rate glove for bicycling or everyday wear. No one would guess it to be an imitation. Its price is 2s. 6d. Chevrette and Mocha gloves, the latter very soft and nice, in grey, tan, etc., are the same price, and gazelle or deer-skin are from 5s. 6d.

### DAINTY GIFTS AT HARRIS'S.

THERE are so many dainty gifts on view at Messrs. J. Harris's, 25 Old Bond Street, that it is a difficult matter to know, not so much what to mention, as what to leave out for lack of space; and I cannot, I think, do better than advise nurses on the look-out for pretty and inexpensive Christmas presents for their friends to send for a catalogue, or, if possible, pay a personal visit of inspection. One feature of this firm is that almost everything shown in its finished state has its counterpart traced and begun ready for working; and the value of the gift may thus be greatly enhanced, provided one has a little leisure and a taste for dainty embroideries. One of the latest novelties is the "stitch-in-time" work board, made in art linen, and containing pincushion, needlecase, packets of needles, scissors, cottons, etc., which would form a most useful present to a busy nurse. Then I noticed a large cosy, for putting over a hot-water can, and this, like many other articles, had a suitable motto worked in thread. Very warm and cosy-looking, too, are the bags for hot-water bottles, or bricks, made of white woollen material, and with the words, "Hot Bottle," or "Hot Brick," traced on them. These are 1s. 6d. traced, while the cost of the completed case is 2s. 6d. Hat-pin cases, to take on a journey and to look pretty on the dressing-table, embroidered cases for autographs, telegrams, snapshots, picture post-cards, blotters, and for the new counterfoils of postal-orders, are among the pretty things shown me by the lady manageress, while the little scent sachets and diaries might very well replace the Christmas card, when a nice but inexpensive souvenir is wanted. Then there are boudoir cushions, table-centres, effectively appliquéd with oranges and apples, and, for the nursery, a blue rug with animals cut out in white swans-down and buttonholed round.

I was particularly pleased with the "dress garnitures," consisting of appliqué linen embroideries on cloth or serge, and with the new stock collars and ties, worked in linen or lawn in various delicate shades, which are in such favour at present. These are very easily worked, and only cost a shilling or so, including the silk for embroidery. Messrs. Harris have showrooms also at 33 King Street, Manchester, and 89 Corporation Street, Birmingham.

### USEFUL GIFTS AT MESSRS. EGERTON BURNETT'S.

ANOTHER large box of patterns has reached me from Messrs. Egerton Burnett. Amongst other things there are some really handsome Scotch tartan travelling rugs, the very sight of which reminds one of those long, cold journeys to a distant case which the private nurse has so frequently to undertake, and there could hardly be a more suitable Christmas present for her than one of these. They range from 5s. 11d. to 1 guinea in price, and if your friend is a Scotswoman, you may perhaps be able to hit upon her special "clan" colouring and design, a delicate attention which would certainly enhance the gift. The reversible tweeds, beautifully warm and thick, are eminently suitable for golf capes or extra wraps; they are in grey, brown, blue, or black, and there is a heather mixture, of which the inner side is a pretty check of blue and red. For gifts to one's poorer neighbours (or may I suggest for our own Christmas Distribution?) real Welsh shawls are most suitable. These are fringed, all wool, and very warm, just the thing for a patient leaving hospital. Dark blue or grey serge under-skirts are also very suitable for such a purpose; they are from 1s. 9½d., ready made up. Or there is a special "charity parcel," containing many yards of dress and skirt material, a wool shawl, a man's shirt, four pairs of women's stockings, and two of men's; while another 4s. 6d. will purchase in addition cardigan jackets, a striped bed rug, and two under-skirts. Both parcels are generously made up, and have been supplied to Royal commands. My readers already know that a large range of washing materials for nurses' dresses is always kept in stock, for many of the uniforms required for probationers' and nurses' indoor wear come from Wellington; these are well known for their durability as well as their moderate price. Accessories, such as apron linen, collars and cuffs, pocket-handkerchiefs, plain or embroidered, stockings, gloves, corsets, and underclothing, and knitted wool vests, can all be had from this firm, which also supplies travelling bags, hold-alls, etc. Nurses should write for patterns and price lists.

### SCENTS AND SOAPS.

At the show-rooms of the Erasmic Company, 117 Oxford Street, are many dainty gifts suitable for Christmas. The perfumes, which can be bought of all the leading chemists, are put up in cut-glass bottles from one shilling, whilst those known as the "Erasmic," the "Gaiety," and "Dinna Forget" are from eighteenpence upwards. There is a charming variety of bottles to choose from. Perfume presentation caskets are from half-a-crown, and any of these would make a welcome present to lovers of scents. Then there are toilet soaps, which I can recommend from personal experience. They are soft and creamy to use, and would be especially acceptable to those whose hands suffer from rough skin. There are various kinds, such as the "Peerless Erasmic," the "Elite Erasmic," "La Belle Erasmic," and "Erasmic de Luxe," and of course the prices vary, too; but they are by no means exorbitant, and a box of three tablets at one shilling or eighteenpence would make a useful as well as an inexpensive Christmas gift to a friend. Then for one's brothers there are luxurious shaving soaps and creams made up in fancy ways at equally moderate prices. The window of the Oxford Street show-room has just been specially dressed for Christmas, and the managers invite inspection.

### 4711 EAU DE COLOGNE.

The shops are full of attractive objects to tempt those in search of presents for their friends. But most that we see are pretty trifles of very passing interest. The purchasers are much wiser who turn to the ever welcome gift of a bottle of the unrivalled 4711 Eau de Cologne, sold at Messrs. Mühlens' depôt, 162 New Bond Street. Such a present pleases friends of either sex. Messrs. Mühlens are constantly introducing novelties in perfumes. On this occasion we call to our readers' notice the delightful extract violetta graziella, and the sachets made of the same perfume. Both are most attractive.

### THE "JEWEL" FOUNTAIN PEN.

It is difficult to find suitable gifts at all times for our male friends. The present of a fountain pen will always be acceptable. The "Jewel" is a reliable and inexpensive variety, sold at 102 Fenchurch Street. It has a good reservoir, a duplex feeder, and a neat and flexible little pen-nib of any breadth in the point which is preferred. A fountain pen is an invaluable companion.

### Appointments.

City Infectious Hospital, Hereford.—Miss B. M Stafford has been appointed matron. She was trained at the Sanitary Hospital, Bournemouth, and has since been nurse at the Sanatorium, Eastbourne, staff nurse and sister at the City Hospital, Coventry, assistant nurse at the City Hospital, Grafton Street, Liverpool, and staff nurse at the Convalescent Home, Gildersome, near Leeds.

Colonial Nursing Association.—Miss A. E. Ball has been appointed nurse in the Nursing Home, Freetown, Sierra Leone. She was trained at Mill Road Infirmary, Liverpool where she was afterwards sister. As a member of the Army Nursing Service Reserve she did duty at Aldershot and in South Africa for upwards of two years.

Eastey Union Infirmary.—Miss H. Jones has been appointed head nurse. She was trained at the Stockport Union Infirmary and was also staff nurse and sister in the same institution. She has been pupil night nurse in the maternity wards at Lambeth Workhouse. She holds the L.O.S. certificate.

Ipswich Infectious Diseases Hospital.—Miss F. K. Monkhouse has been appointed matron. She was trained at St. George's Hospital, London, and the South-Eastern Fever Hospital, New Cross. She has since been matron of Darwen Borough Fever Hospital.

Royal Hospital for Sick Children, Edinburgh.—Miss Clementina Kemp has been appointed sister. She was trained at the Royal Hospital for Sick Children, Edinburgh, and the Western Infirmary, Glasgow. She has since been charge nurse at the Fountain Fever Hospital, Tooting, S.W., has served in South Africa as a member of the Army Nursing Reserve, and lately she has been attached to the Scottish Association of Trained Nurses, Alva Street, Edinburgh.

Royal Ophthalmic Hospital, City Road, London.—Miss E. M. Cooke has been appointed matron. She was trained at the Taunton and Somerset Hospital, where she has since been night superintendent. She has also been staff nurse at St. George's Hospital, London, and out-patient sister at the Royal London Ophthalmic Hospital.

Warrington Isolation Hospital.—Miss E. Lees has been appointed matron. She was trained at St. Thomas's Hospital, London, and for the last six months has been assistant matron at Warrington Isolation Hospital.

### Where to Go.

Adelphi Theatre, Strand.—Dress Rehearsal of "Little Hans Andersen," Tuesday, December 22nd, at 2 p.m. Seats on sale at double the usual prices, the proceeds to be devoted to the Hospital for Sick Children, Great Ormond Street, Bloomsbury, W.C.

### Death in Our Ranks.

Miss Alice Gertrude Bailey, who succumbed to an attack of typhoid fever at Guy's Hospital on the 23rd ult. entered the hospital as a probationer in February last, and was in charge of a case in Clinical ward immediately before her illness. A memorial service was held in the hospital chapel on the Thursday following her death, at which members of the medical staff, the matron, and some hundred nurses, as well as relatives of the deceased, were present. The body was afterwards conveyed to Leicester, the residence of Miss Bailey's father, Mr. Edward Bailey, M.R.C.S. Among the many wreaths was one composed of violets and white flowers from the nursing staff; and tributes were also sent by the clinicals of the hospital, the sisters and nurses of Clinical and Esther wards, Sisters Enid and Ada (coaches in the preliminary training school), and by fellow pupils

### Everybody's Opinion.

[Correspondence on all subjects is invited, but we cannot in any way be responsible for the opinions expressed by our correspondents. No communication can be entertained if the name and address of the correspondent are not given as a guarantee of good faith, but not necessarily for publication. All correspondents should write on one side of the paper only.]

### JUNIUS S. MORGAN BENEVOLENT FUND.

THE Secretary of the Royal National Pension Fund for Nurses asks us to intimate the receipt of a third annual remittance of £1 from "A Hospital Sister," who requests that it shall be acknowledged through the columns of THE HOSPITAL.

### DISCIPLINE AT CHARING CROSS HOSPITAL.

MISS LUCY RAE writes from 74 Lissenden Mansions, Highgate Road, N.W.: Some extraordinary reports are current respecting the hours off duty of the sisters and nurses in Charing Cross Hospital. Will you kindly permit me, as one who was trained in Charing Cross, and who worked in that institution for six years, to mention a few reliable facts? The nursing staff were off duty between the hours of 10 A.M. and 8 P.M. only. Anyone who desired to have her leave extended beyond the hour of 8 P.M. was compelled to ask the lady superintendent to grant the indulgence. This rule was rigidly enforced up to the year 1902.

### THE ANGLO-AMERICAN NURSING HOME AT ROME.

SIR RENNEL RODD, Secretary of the British Embassy at Rome, writes: In common with many others in Rome who take a keen interest in the welfare of our Anglo-American Nursing Home, I have been much distressed to see that a persistent campaign against that institution has been inaugurated in THE HOSPITAL by certain anonymous correspondents. I have too much experience of Anglo-Saxon communities abroad to hope that absolute harmony will anywhere prevail in the management of their local institutions; but is it too much to ask that, if certain dissentient members elect to appeal from the verdict of the majority to the public press at home, they should at least take a minimum of trouble to verify their facts before committing themselves to disparaging assertions? On p. 83 of THE HOSPITAL of November 7th, there appears a letter, or extract from a letter, signed "A Resident in Rome," and dated October 29th, containing the following statement: "a free-bed case was refused last week because there was only one nurse in the home at the time." As this assertion was contrary to my own experience, I investigated the matter further, and found that the statement was as incorrect as the reason alleged for it. Neither in the week preceding October 29th, nor at any time since the home has been open, has a free-bed patient ever been refused. One was as a matter of fact admitted on October 26th. It is possible that the postponement of this patient's admission for two days, in agreement with the doctor in charge of the case, may have misled your correspondent. It cannot, however, excuse the recklessness of the assertion. With regard to the reason assigned for the alleged refusal, the facts are as follows. On the reopening of the home after the summer season, the nurses arrive in batches, as they are likely to be wanted. Three arrived in Rome on October 1st, and a fourth on the following day. Two more came on October 31st, and three others on November 2nd. The statement of "A Nurse" in THE HOSPITAL of November 17th, which your correspondent traverses to the effect that five of the old nurses had returned, meant, of course, had accepted engagements to return. Four nurses were, however, actually in Rome at the time referred to. In placing this information at your disposal I should like to add that I have been most reluctant to enter into this controversy, which has however been forced on my notice, and if I have done so on this occasion it is in the hope that this simple answer to the latest criticism offered by an anonymous correspondent will not prolong a discussion which in my opinion can lead to no good end.

### PRIVATE NURSING IN CALIFORNIA.

MRS. HELEN C. SEXTON writes from Visalia Sanitarium, California, U.S.A., under date of November 6th, 1903: Some time ago I advertised in your paper for a nurse wishing to take up private nursing in California. I received 51 replies. Of these 25 put insufficient postage and only two sent stamps for a reply, yet asked a number of questions. Other replies came a few days later, which, having insufficient postage, the doctor advised my not taking in, as each letter cost us 5d. and in some cases as much as 2s. and more, as testimonial copies were enclosed. By his advice I write to you, as many nice nurses, by just such a little act of carelessness, are likely to miss a good appointment abroad.

### TRAINING UNDER THE ROYAL ARMY MEDICAL CORPS.

"AUBREY" writes: In answer to your correspondent "Trained Nurse," I beg to state that the assertion of THE HOSPITAL to the effect that the National Hospital is the only training school for male nurses in Great Britain is a correct one. The National nurse spends his whole time in the wards, and he is thoroughly instructed in the theory and practice of nursing, besides catheter work, massage, and the application of medical electricity. The Royal Army Medical Corps orderly has to attend several parades and drills a day, and a man cannot be expected to take an interest in his work if, when in the midst of attending to a patient, he has to run away to parade. A military orderly is excellent at stretcher-bearing and "first aid" work, but he cannot accurately call himself a trained nurse. If the training of Royal Army Medical Corps orderlies is a complete one, why should they come to the National Hospital for a training? There are four probationers at the National Hospital at the present time who have had military experience, one of whom was in the Royal Army Medical Corps for $6\frac{1}{2}$ years, attaining the rank of corporal.

"ESPRIT DE CORPS" writes from the National Hospital, Queen Square, Bloomsbury: In reply to "Trained Male Nurse" whose letter appeared in THE HOSPITAL for the 28th ult., I beg to differ from his opinion as to the value of the training of the Royal Army Medical Corps. I do not doubt for one moment his success as a nurse, but as I have had some experience as a member of the Royal Army Medical Corps on active service I can honestly say there is no "training" in regard to nursing duties. What a man learns in the Royal Army Medical Corps about nursing has to be picked up during his work, and the more intelligent the man, the better he is when he has finished his service. There is certainly no practical teaching of "nursing duties." I do not wish to deprecate the value of the knowledge acquired by the men of the Royal Army Medical Corps, but it is certainly not sufficient as compared with that to be obtained at the "National Hospital." We have now among the male nurses at this hospital no less than four out of nine who have served for varying periods, either in or attached to the Royal Army Medical Corps, and at least two have been non-commissioned officers. Further comment is unnecessary, as these few facts speak for themselves.

### Wants and Workers.

"THE HOSPITAL."—Will any nurse kindly send THE HOSPITAL on the Monday after publication? Postage prepaid. Nurse, care of 171 High Road, Lee, S.E.

WILL any matron or nurse kindly send THE HOSPITAL, when finished with, every week, for which postage will be sent in advance? Address Nurse Osborne, 5 York Villas, Portland Road, Bishop Stortford, Herts.

MACKINTOSH.—A trained nurse would be very grateful for cast-off mackintosh or warm cloak. Nurse Mary, 1 Askew Crescent, Shepherd's Bush, W.

### Notes and Queries.

**FOR REGULATIONS SEE PAGE 135.**

*Abroad.*

(96) Will you kindly give me the names of one or two good nursing institutions on the Continent?—*C. H. H.*

The English Nurses' Home, Villa Albany, Biarritz; the Davos Invalids' Home, Davos Dorf, Switzerland; The Association of Trained Nurses and Masseuses, 7 Via Rondinelli, Florence; the Nice Nursing Institute, Villa Pilatte, Avenue Désambrois, Nice; the English Nurses' and Medical Home, Sunny Bank, Via Borgo Pescia, San Remo, etc. We give these from our list, we do not undertake to recommend.

*Queen's Nurses.*

(97) Will you kindly tell me to whom to apply for admission to Queen Victoria's Jubilee Institute for Nurses?—*E. W.*

Apply the General Superintendent, 120 Victoria Street, S.W.

*List of Nursing Homes.*

(98) Will you kindly tell me how I can get a list of nursing homes and institutions taking in patients and otherwise?—*G. L. F.*

You will find a short list of institutions receiving mental and consumptive patients in "Burdett's Hospitals and Charities," which can be obtained from the Manager of the Scientific Press, 28 Southampton Street, Strand, W.C. But there is no authentic list published of the innumerable private institutions and homes.

*Children's Nurse.*

(99) Will you kindly tell me of an institution where ladies can be trained as children's nurses?—*A. H. G.*

The Norland Institute, 10 Pembridge Square, W.; the Liverpool Ladies' Sanitary Association, 8 Sandon Terrace, Liverpool; and the Convalescent or Permanent Home, Uplands, Loughton, Essex.

*Fees.*

(100) Will you please inform me if it is customary to stop a nurse's fee whilst she is having a week's rest when attending a permanent case, no substitute having been employed in her absence?—*F. M. G.*

It depends entirely upon the circumstances of the case. If the employer grants a holiday, it is usual to continue the nurse's salary.

*Colonial Nursing Association.*

(101) I should be grateful if you would give me the address of the Colonial Nursing Association.—*Doris.*

Imperial Institute, S.W.

*Home.*

(102) Will you kindly inform me if there is a home where a housemaid, suffering from rheumatoid arthritis, could be received free of charge for a time?—*M. H.*

The Devonshire Hospital and Buxton Bath Charity, Buxton, seems most suitable.

*Hospital Training.*

(103) I should be very glad if you could tell me what hospital in Liverpool would be likely to accept me as probationer. I am unable to pay any sum to enter, and I should like just a little wage as soon as possible.—*E. S.*

Write and ask the Superintendent of Nurses, the Workhouse Infirmary, Brownlow Hill, Liverpool, for a form of application. Probationers do not have to pay a premium at this infirmary, and the salary for the first year is £10.

*Military Nursing Service.*

(104) Please tell me to whom to apply for particulars of Queen Alexandra's Imperial Military Nursing Service?—*A. P. B.*

The Under-Secretary of State, War Office, Pall Mall, S.W.

### Important Nursing Textbooks.

"The Nursing Profession: How and where to Train." 2s. net 2s. 4d. post free.

"A Handbook for Nurses." By Dr. J. K. Watson. 5s. net; 5s. 4d. post free.

"Practical Guide to Surgical Bandaging and Dressings." By Wm. Johnson Smith, F.R.C.S. 2s. post free.

"The Nurses' Dictionary of Medical Terms and Nursing Treatment." By Honnor Morten. 2s. post free.

"The Human Body: its Personal Hygiene and Practical Physiology." By B. P. Colton. 5s. post free.

**"Art of Feeding the Invalid." (Popular Edition). 1s. 6d. post free.**

"On Preparation for Operation in Private Houses. By Stanmore Bishop, F.R.C.S. 6d. post free

### For Reading to the Sick.

THE HEAVENLY SOWER.

Within a hallow'd acre
He sows yet other grain,
When peaceful earth receiveth
The dead he died to gain;
For though the growth be hidden,
We know that they shall rise;
Yea even now they ripen,
In sunny Paradise.
O summer land of harvest,
O fields for ever white
With souls that wear Christ's raiment,
With crowns of golden light!

One day the heavenly Sower
Shall reap where He hath sown,
And come again rejoicing,
And with him bring his own;
And then the fan of judgment
Shall winnow from His floor
The chaff into the furnace
That flameth evermore.
O holy, awful Reaper,
Have mercy in the day
Thou puttest in thy sickle,
And cast us not away.

*St. Hill Bowne.*

"He that believeth in me, though he were dead, yet shall he live."

Do such words as these mean only that we shall rise again in the resurrection at the last day? Surely not, our Lord spoke them in answer to that very notion.

"Martha said to Him, I know that my brother shall rise again, in the resurrection at the last day. Jesus said unto her, I am the Resurrection and the Life;" and then showed what He meant, by bringing back Lazarus to life, unchanged, and as he had been before he died.

Surely if that miracle meant anything, if these words meant anything, it meant this: that those who die in the fear of God and in the faith of Christ do not really taste death; that to them there is no death, but only a change of place, a change of state; that they pass, at once and instantly, into some new life, with all their powers, all their feelings, unchanged—purified doubtless from earthly stains, but still the same living, thinking, active beings which they were here on earth. I say active, the Bible says nothing about their sleeping till the Day of Judgment, as some have fancied. Rest they may, rest they will, if they need rest. But what is the true rest? Not idleness, but peace of mind. To rest from sin, from sorrow, from fear, from doubt, from care—this is the true rest. Above all, to rest from the worst weariness of all—knowing one's duty, and yet not being able to do it. That is true rest; the rest of God, Who works for ever, and yet is at rest for ever; as the stars over our heads move for ever, thousands of miles each day, and yet are at perfect rest, because they move orderly, harmoniously, fulfilling the law which God has given them. Perfect rest, in perfect work; that surely is the rest of blessed spirits, till the final consummation of all things, when Christ shall have made up the number of His elect.

*C. Kingsley.*

## Figures and Tables

**Figure f1:**
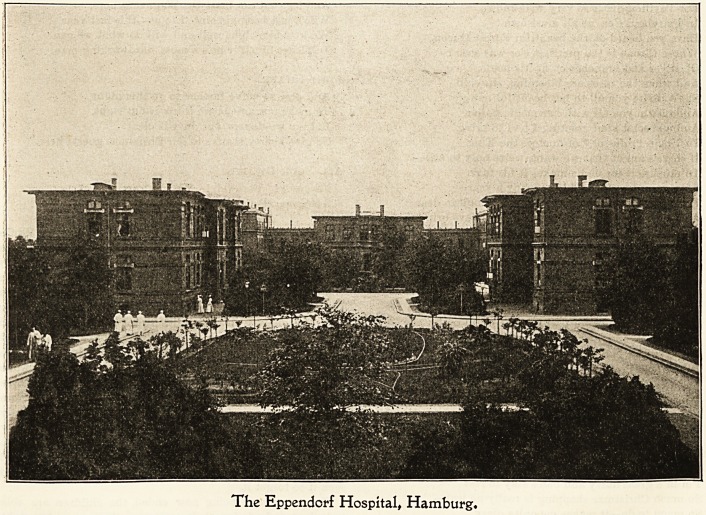


**Figure f2:**
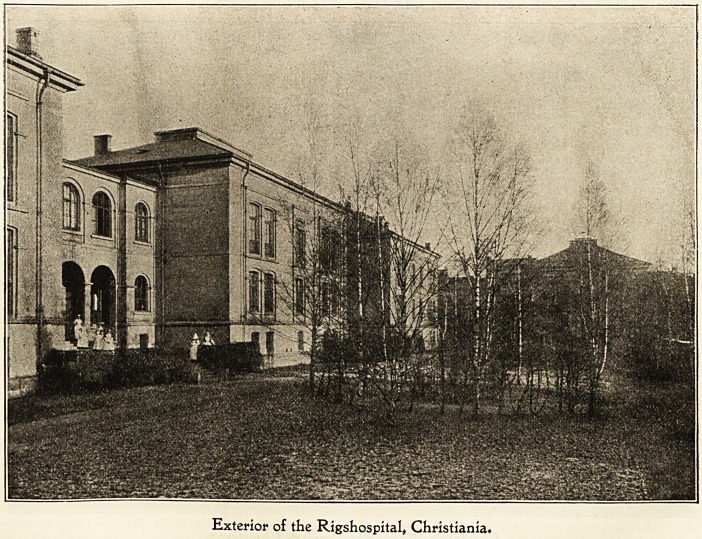


**Figure f3:**
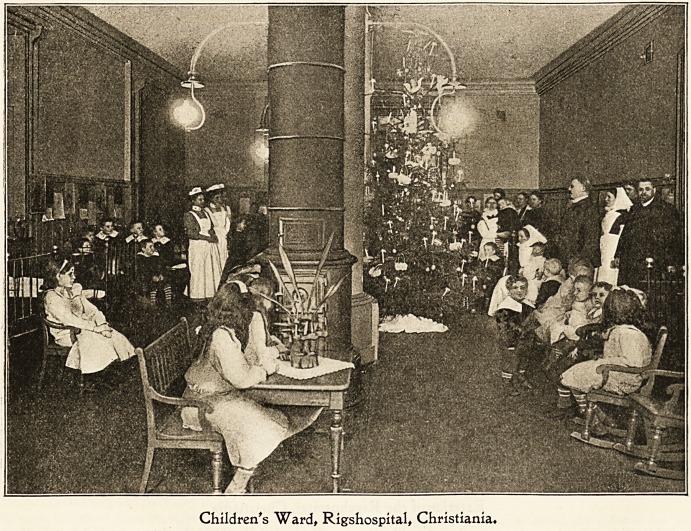


**Figure f4:**
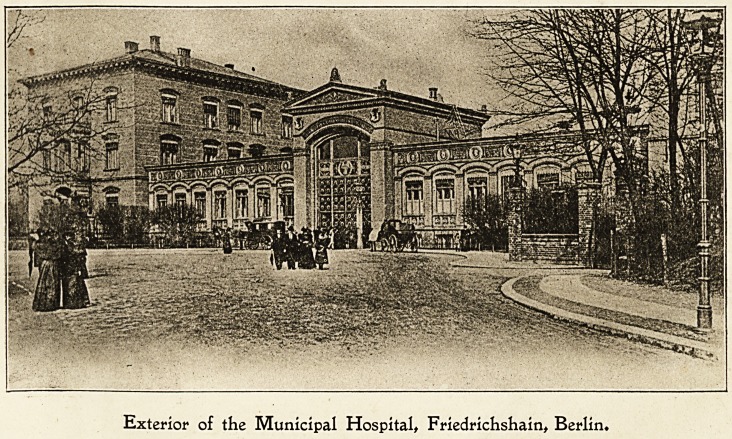


**Figure f5:**
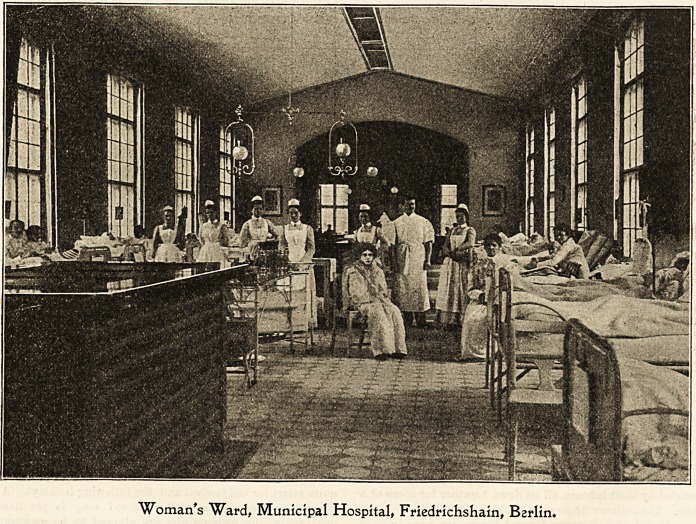


**Figure f6:**
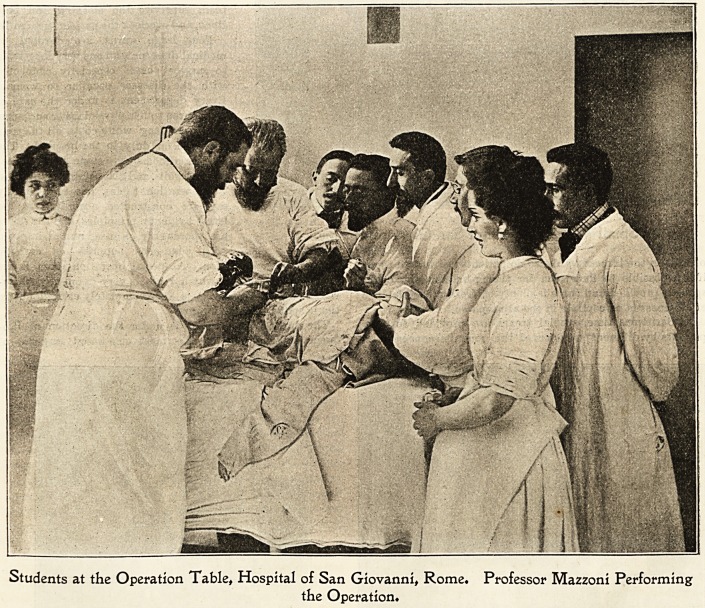


**Figure f7:**
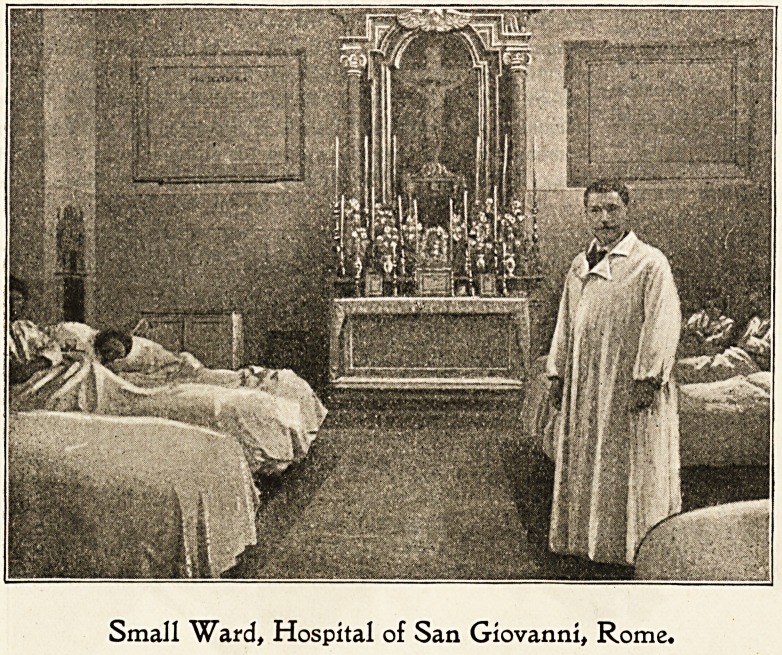


**Figure f8:**
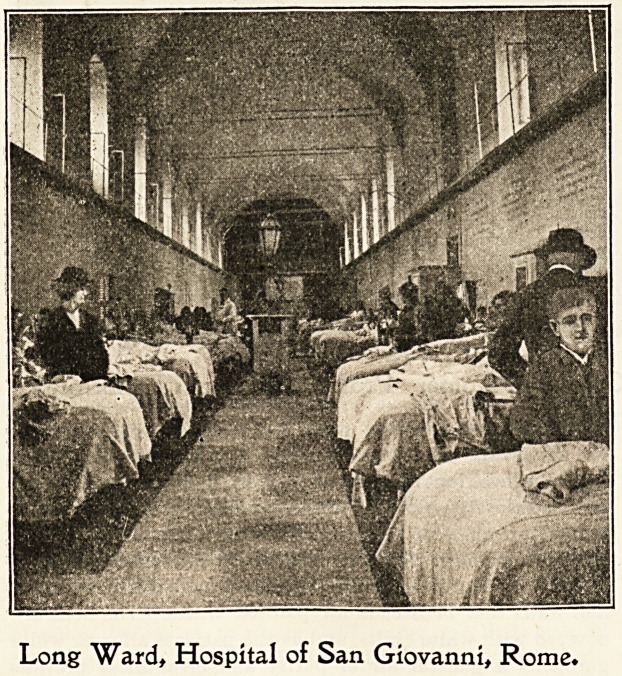


**Figure f9:**
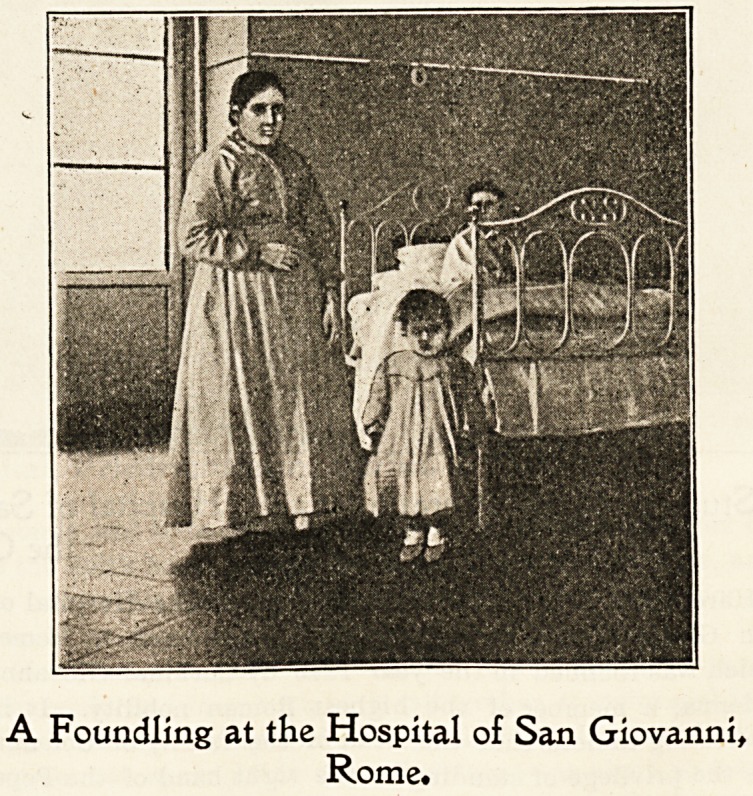


**Figure f10:**
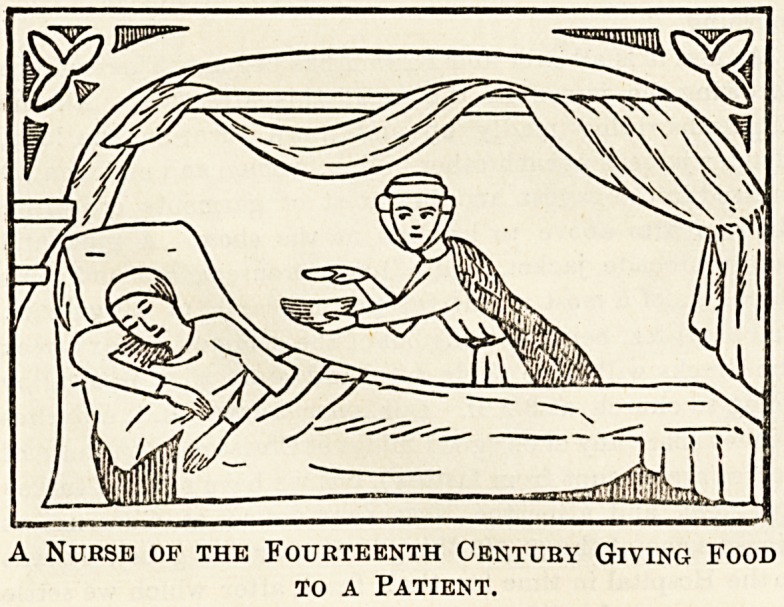


**Figure f11:**
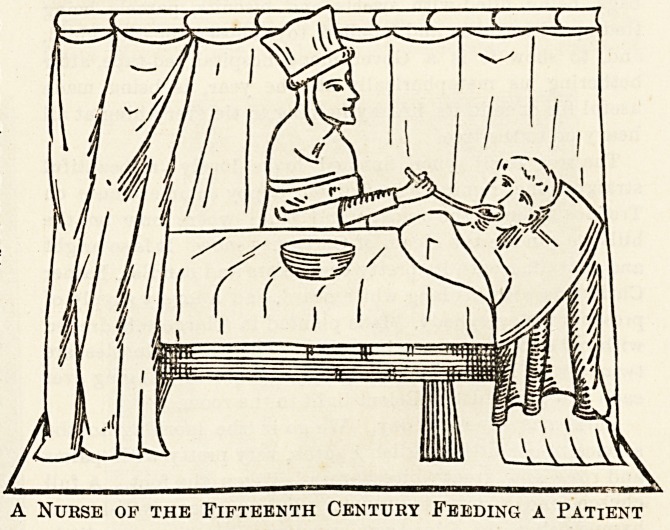


**Figure f12:**
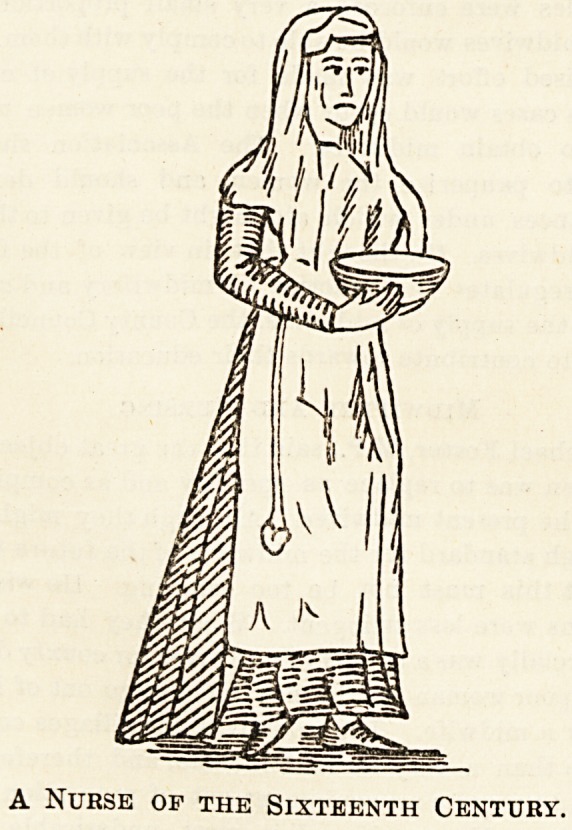


**Figure f13:**
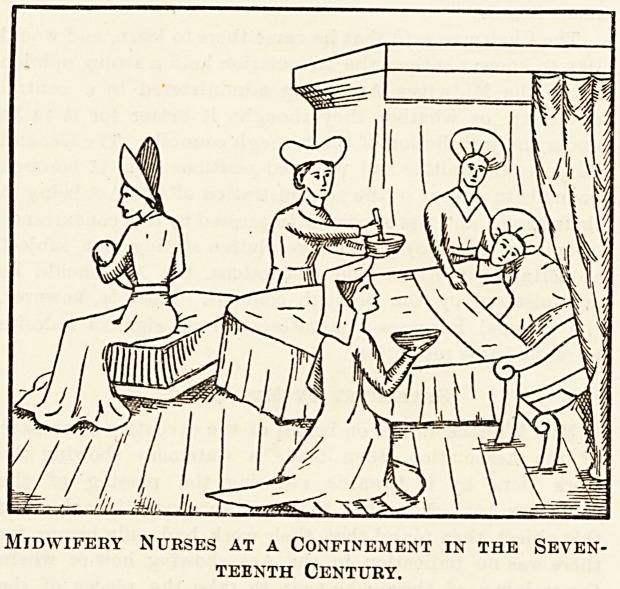


**Figure f14:**